# An Advanced Adhesive Electrolyte Hydrogel Intended for Iontophoresis Enhances the Effective Delivery of Glycolic Acid Via Microbeads

**DOI:** 10.3390/gels11090682

**Published:** 2025-08-26

**Authors:** Mariia Kazharskaia, Yu Yu, Chenguang Liu

**Affiliations:** 1 College of Marine Life Sciences, Ocean University of China, No.5 Yushan Road, Qingdao 266003, China; marikazharskay@mail.ru (M.K.); yuyu@youdo-bio.com (Y.Y.); 2 Qingdao Youdo Bioengineering Co., Ltd., No.175 Zhuzou Road, Qingdao 266101, China

**Keywords:** electrolyte hydrogel, microbead, glycolic acid, iontophoresis

## Abstract

This study presents an innovative iontophoretic delivery system for glycolic acid (GA) based on polysaccharide microbeads embedded within an electrolyte hydrogel. The mi-crobeads, fabricated using a peristaltic pump, exhibited a uniform morphology with an average diameter of 1078 ± 140.38 μm and were successfully integrated into a hydrogel matrix (thickness: 4542.55 ± 337.24 μm). Comprehensive physicochemical characterization (FT-IR, XRD, SEM) confirmed effective component integration. The hydrogel demonstrated optimal mechanical properties with a tensile strength of 0.02 ± 0.003 MPa and reliable adhesion to various substrates, while maintaining excellent self-healing capabili-ties—post-repair conductivity remained sufficient to power an LED indicator. The material demonstrated favorable conductivity under various storage conditions while maintaining non-cytotoxic properties. Notably, microbead incorporation preserved electrochemical performance, as demonstrated by stable behavior in cyclic voltammetry using an Ag/AgCl reference system. Iontophoretic testing revealed significantly enhanced glycolic acid delivery at −1.0 V com-pared to passive diffusion. The system, combining PVA, PAA, alginate, [Bmim]BF_4_, and E. prolifera polysaccharides with gellan gum, shows strong potential for advanced cosmetic dermatology applications requiring precise active ingredient delivery.

## 1. Introduction

Aging poses a significant challenge in contemporary dermatology, driving the need for simple yet effective materials and devices for anti-aging therapies [1]. Recent advances in combating skin aging have led to the development of various delivery systems—including gels, emulsions, and cream—that enhance targeted skin rejuvenation [2,3,4]. Among these, electrically assisted transdermal delivery systems combining active ingredients with stimulation mechanisms show particular promise [5]. Iontophoresis has emerged as an especially promising non-invasive technique that utilizes low-intensity electrical currents to facilitate transdermal transport of active compounds while offering superior patient compliance and dosing control compared to oral or parenteral administration [6].

Conductive hydrogels demonstrate exceptional potential for transdermal applica-tions due to their dual functionality of electrical responsiveness and drug-loading capaci-ty, enabling their use as smart electrode patches [7,8]. These hydrogels have emerged as particularly promising materials for iontophoretic patches because of their unique combi-nation of electrical conductivity and biocompatibility. Through the incorporation of elec-tronically conductive polymers like polyaniline, flexible electrode, or PEDOT, they enable more efficient and stable drug delivery under electric fields [9,10,11].

In iontophoretic applications, conductive hydrogels minimize electrode polarization, ensure a uniform current distribution, and maintain consistent drug release rates [12]. Additionally, their flexible and adhesive nature allows for conformal contact with the skin, reducing interfacial resistance while improving patient comfort. Notably, conductive hydrogel-based patches have demonstrated enhanced transdermal delivery of drugs like viologen and lidocaine, with precise control over dosage and release kinetics [13,14]. While conductive hydrogels demonstrate excellent current distribution properties, several practical challenges remain that limit their widespread clinical adoption. The incorporation of conductive fillers like carbon nanomaterials or metal particles often increases hydrogel stiffness, potentially compromising conformability to skin surfaces [15,16]. However, these composite materials also raise concerns about long-term biocompatibility, as par-ticulate leaching could trigger inflammatory responses in chronic applications. Further-more, the electronic conductivity mechanism in these materials does not inherently im-prove actual ion transport—the fundamental requirement for effective iontophoresis.

These limitations reinforce the continued preference for optimized electrolyte hydrogels in most therapeutic applications. Their pure ionic conductivity mechanism directly facilitates drug transport without requiring conductive fillers. The absence of particulate components ensures proven biocompatibility, while their softer mechanical properties maintain better skin contact [17,18,19]. More importantly, ionic liquids (ILs) have been incorporated as electrolytes due to their exceptional properties, including high conductivity, negligible vapor pressure, non-flammability, wide electrochemical window, and thermal stability [20]. Recent advances aim to bridge this gap by developing hybrid systems that combine the ionic conductivity of traditional hydrogels with the electronic conductivity of nanomaterials. Such innovations could revolutionize iontophoretic therapy.

**Scheme 1:**
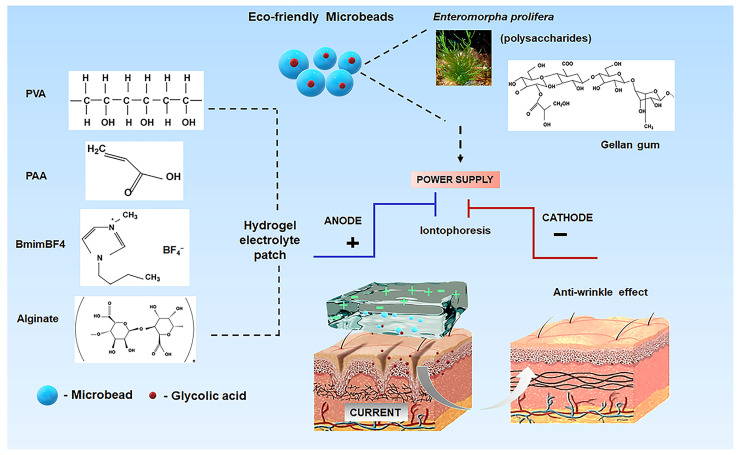
Schematic representation of polysaccharide microbeads integrated within a PVA-PAA-alginate electrolyte hydrogel for iontophoresis devices, demonstrating the controlled release mechanism of glycolic acid.

Here we present a novel electrolyte platform using polysaccharide microbeads as a delivery source for glycolic acid—a well-established cosmetic active ingredient—within a specially designed electrolyte hydrogel matrix for potential iontophoretic applications (Scheme 1). The synergistic combination of components including polyvinyl alcohol (PVA), polyacrylic acid (PAA), and alginate, along with the specific integration of [Bmim]BF_4_, significantly enhances hydrogel conductivity. This synergistic system represents an advanced platform for controlled compound delivery. By combining iontophoretic enhancement with sustained glycolic acid release, this technology offers a promising alternative to conventional topical treatments, with potential for improved skin rejuvenation outcomes.

## 2. Results and Discussion

### 2.1. Characterization of Polysaccharide Microbeads and Electrolyte Hydrogel

To obtain the microbeads, we employed a specialized peristaltic pump method (Scheme S1). The peristaltic pump was first combined with a nozzle configuration featuring 3.6 mm inlet and 2.5 mm outlet diameters. Subsequently, 10 mL of polysaccharide solution was pumped at a flow rate of 150 mL/min through the nozzle and dispensed into a 10% CaCl_2_ continuous phase. Polysaccharide microbeads containing glycolic acid were also produced using this pump system. Finally, these two types of microbeads were compared through subsequent characterizations.

**Figure 1:**
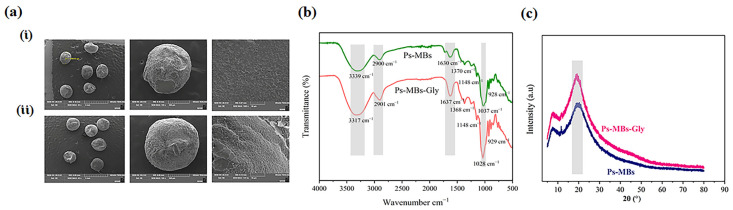
(**a**) Scanning electron microscopy images of (*i*) Ps-MBs and (*ii*) Ps-MBs-Gly microbeads; (**b**) Fourier-transform infrared spectra and (**c**) X-ray diffraction patterns of Ps-MBs and Ps-MBs-Gly microbeads.

SEM analysis (Figure 1a) confirmed that glycolic acid incorporation did not signifi-cantly alter microbead morphology, with both Ps-MBs and Ps-MBs-Gly maintaining rough surfaces and comparable average diameters (1065 ± 12 μm and 1076 ± 15 μm, respectively). High-resolution imaging revealed continuous, non-porous surfaces without structural defects, demonstrating excellent matrix stability during active component encapsulation.

FT-IR analysis of microbeads (Figure 1b) confirmed structural integrity in both for-mulations, with broad peaks at 3339 cm^−1^ (Ps-MBs) and 2900–2901 cm^−1^ (COOH) [21]. A 22 cm^−1^ redshift (3317 cm^−1^) in Ps-MBs-Gly suggests hydrogen bonding between polysaccharide hydroxyls and glycolic acid carboxyls. This was further supported by changes in the carbonyl region (1637 cm^−1^ C=O stretch), indicating glycolic acid’s influence on gellan gum carboxylates [22]. The fingerprint region (1037–1028 cm^−1^ and 928–929 cm^−1^) showed little variation, confirming an intact polysaccharide backbone after loading [23]. Both microbead types retained key peaks (3500 cm^−1^, 3317 cm^−1^, 2900–2901 cm^−1^), proving their consistent chemical functionality. XRD analysis (Figure 1c) maintained crystallinity near 20°, with a 2° shift to 18° in Ps-MBs-Gly indicating altered molecular packing from glycolic acid–polysaccharide interactions. FT-IR, SEM, and XRD analyses confirmed the uniform glycolic acid distribution in the polysaccharide matrix while preserving the microbead structure and enabling controlled interaction modification. These results demonstrate the microbeads’ ideal properties for advanced hydrogel delivery systems, where structural integrity during loading is vital for controlled release.

Swelling tests in buffer solutions (2, 4, and 10 days; Table S1) showed that mi-crobeads maintained structural integrity across pH levels (Figure S1). Imaging revealed slight swelling by day 10 (0.33 ± 0.02 g/g; Table S1). Crucially, core integrity remained in-tact, demonstrating strong interfacial adhesion between gellan gum and algal polysaccha-ride chains [24]. SEM analysis (Figure S2) confirmed the structural stability, with an un-changed morphology after pH buffer extraction. Glycolic acid release (Figure S3) showed similar yields at pH 5.5 (39.1 ± 0.5%, SEM = 0.25) and pH 7.4 (38.1 ± 0.27%, SEM = 0.14) versus controls (45.8 ± 0.15%, SEM = 0.075 and 39.4 ± 0.15%, SEM = 0.075), indicating minimal diffusion resistance from the microbead matrix. These results demonstrate the microbeads’ stability for glycolic acid delivery, enabling hydrogel integration.

The microbeads prepared using a peristaltic pump (Scheme S1) were evaluated for glycolic acid release kinetics at pH 5.5 and 7.4 (Figure S4). The study demonstrated that after 210 min at pH 5.5, larger microbeads (1141 ± 11 μm and 945 ± 45 μm) exhibited higher glycolic acid release rates of 36.68 ± 0.09% and 36.58 ± 0.04%, respectively, compared to smaller microbeads (750 ± 45 μm and 652 ± 74 μm), which released 24.36 ± 0.08% and 23.16 ± 0.09% glycolic acid. A similar trend was observed at pH 7.4, where larger microbeads showed releases of 44.05 ± 0.12% and 43.72 ± 0.10%, while smaller ones released 25.37 ± 0.16% and 22.85 ± 0.11%. This behavior can be attributed to the fact that larger microbeads likely incorporate more glycolic acid during synthesis due to their greater matrix volume, providing higher initial loading capacity. Furthermore, their polysaccharide network may possess a more open porous structure that facilitates the easier diffusion of glycolic acid molecules. In contrast, smaller microbeads not only contain less initial glycolic acid due to their reduced volume but may also feature a denser matrix structure that creates greater diffusion resistance. Additionally, the higher surface-area-to-volume ratio of smaller microbeads could lead to faster initial surface hydration and the formation of a more compact gel layer that further hinders release. The increased release at pH 7.4 compared to pH 5.5 for larger microbeads suggests that the deprotonation of carboxyl groups under slightly alkaline conditions enhances glycolic acid solubility and mobility through the polysaccharide matrix, an effect that appears less pronounced in smaller microbeads, possibly due to their more restricted internal structure. These findings demonstrate that microbead size represents a critical parameter controlling glycolic acid release kinetics, with larger microbeads offering distinct advantages in terms of both loading capacity and release efficiency under the tested conditions.

Despite variations in the glycolic acid release from microgranules of different sizes, their cumulative release proves sufficient over the studied time period. The obtained data demonstrate the potential of such microgranules, including those embedded in hydrogel matrices, for the development of stable controlled-release systems.

The integrated system’s structural and functional properties are detailed in the next section.

**Figure 2:**
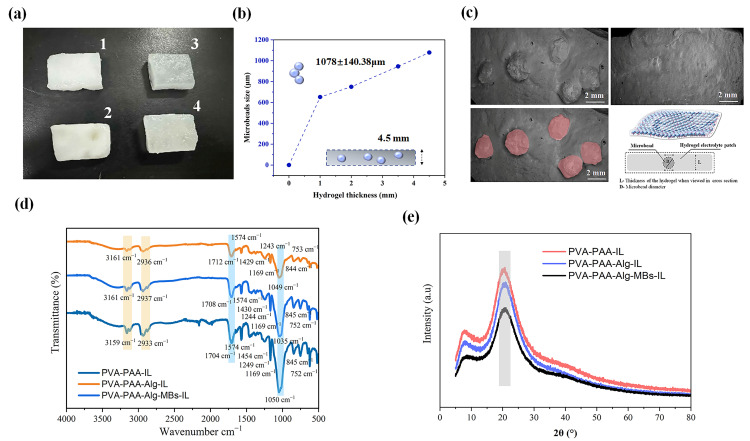
Characterization of hydrogel systems: (**a**) Photographic images of hydrogel variants (1: PVA-PAA-Alg; 2: PVA-PAA-Alg-MBs; 3: PVA-PAA-Alg-IL; 4: PVA-PAA-Alg-MBs-IL). (**b**) Correlation between microbead size distribution (1078 ± 140.38 μm) and hydrogel thickness (4542.55 ± 337.24 μm). (**c**) Cross-sectional SEM images: PVA-PAA-Alg-MBs-IL electrolyte hydrogel with microbeads (highlighted in red, bottom left) and pure PVA-PAA-Alg-IL electrolyte hydrogel (thickness: 3777.45 μm). Schematic (bottom right inset) illustrates microbead distribution in the hydrogel matrix. (**d**) FT-IR spectra of electrolyte nanocomposites (PVA-PAA-IL, PVA-PAA-Alg-IL, PVA-PAA-Alg-MBs-IL). (**e**) XRD patterns of electrolyte nanocomposites. (*n* = 3, mean ± SD).

Figure 2 shows four hydrogel variants: non-electrolyte (1) without and (2) with mi-crobeads, and electrolyte (3) without and (4) with microbeads (Figure 2a). The hydrogels had a uniform 4.5 mm thickness (containing microbeads 1078 ± 140.38 μm; Figure 2b). SEM (Figure 2c) confirmed a homogeneous microbead distribution, with red–pink high-lighting demonstrating uniform integration. This stability is critical for iontophoretic ap-plications like transdermal delivery, where consistent charge transport is essential.

The ionic liquid [Bmim][BF_4_] acts as both dispersant and stabilizer, filling polymer chain gaps while strengthening the hydrogel matrix [25]. This stabilization remains after microbead integration, preserving matrix integrity for reliable electrochemical performance.

The ionic liquid’s dual functionality enhances both material properties and structural stability, enabling robust hydrogels for precise electrochemical and transdermal applica-tions. FT-IR analysis (Figure 2d) compared hydrogel compositions: PVA-PAA-Alg, PVA-PAA-IL, PVA-PAA-Alg-IL, and PVA-PAA-Alg-MBs-IL. The analysis verified compo-nent integration into the PVA-PAA matrix and key polymer–-ionic liquid ([Bmim][BF_4_]) interactions.

As shown in Figure S5, PVA-PAA-Alg hydrogel formulation exhibits structural characterizations by FT-IR analysis. Here, the broad peak at 3278 cm^−1^ indicates extensive hydrogen bonding between hydroxyl groups of PVA and carboxyl moieties of PAA-Alg, evidenced by its significant redshift compared to a pure PVA structure. The coexistence of protonated carboxyl groups at 1704 cm^−1^ (PAA) and ionized carboxylates at 1410 cm^−1^ (alginate) confirms the successful electrostatic interaction between polymer components. Preservation of Alg structural integrity is demonstrated by its fingerprint vibrations at 1025 cm^−1^ (glycosidic linkages) and 799 cm^−1^ (mannuronic acid rings), while the 2919 cm^−1^ (C-H stretch) and 1240 cm^−1^ (C-O ether) peaks reflect undisturbed PVA and PAA backbones. The absence of new peaks verifies the absence of undesirable side reactions, and the relative intensity ratio of 1704 cm^−1^ (COOH) to 1410 cm^−1^ (COO−) establishes blending in this composite system.

Figure 2d demonstrates that the PVA-PAA-IL spectrum exhibited characteristic PVA peaks at 3161 cm^−1^ (O-H stretch) and 1049 cm^−1^ (C-O stretch), alongside PAA peaks at 1712 cm^−1^ (C=O stretch) and 1243 cm^−1^ (C-O-H bend), confirming hydrogen-bonded network formation [26]. The ionic liquid presence was evident from imidazolium ring vibrations (1574 cm^−1^ for C=C/C=N stretching; 844 and 753 cm^−1^ for ring deformations) and the BF_4_− anion signal (1169 cm^−1^ for B-F stretching), demonstrating stable integration into the hydrogel. Upon alginate addition (PVA-PAA-Alg-IL), the PAA C=O stretching band shifted from 1712 to 1708 cm^−1^, indicating electrostatic interactions between PAA carboxyl groups and alginate carboxylates [27]. The FT-IR analysis confirmed alginate incorporation through a new absorption band at 1035 cm^−1^ corresponding to C-O-C glycosidic linkages, while the persistence of characteristic [Bmim][BF_4_] peaks at 1574, 1169, 844, and 753 cm^−1^ demonstrated that ionic liquid maintained its structural integrity within the composite system. The unaffected O-H stretching vibration at 3161 cm^−1^ indicated that alginate integration occurred without disrupting the existing hydrogen bonding network of the hydrogel [28]. These results collectively show that the ionic liquid preserves its molecular structure, alginate selectively modifies the PAA environment through electrostatic interactions while maintaining overall network stability, and the fundamental PVA-PAA hydro-gen-bonded architecture remains intact. This structural preservation ensures the mechanical stability necessary for practical applications in controlled drug delivery and electroactive systems. The detailed FT-IR characterization provides conclusive evidence of successful component integration and elucidates the key interaction mechanisms responsible for the hydrogel’s functional performance.

The FT-IR analysis of the PVA-PAA-Alg-MBs-IL composite revealed additional spectral modifications induced by polysaccharide microbead incorporation. The O-H stretching band exhibited broadening and a slight shift to 3159 cm^−1^, suggesting enhanced hydrogen bonding interactions between the microbeads’ hydroxyl groups and the hydrogel network. A further decrease in the C=O stretching frequency to 1704 cm^−1^ indicated possible esterification or stronger polyelectrolyte complex formation between PAA and polysaccharide components. The appearance of a new peak at 1454 cm^−1^, characteristic of CH_3_ bending vibrations, confirmed successful microbead integration, while the complex C-O stretching region around 1050 cm^−1^ reflected overlapping contributions from PVA, alginate, and microbead polysaccharides [29]. These spectroscopic changes demonstrate the microbeads’ successful incorporation and their molecular-level interactions with the hydrogel matrix, which are crucial for maintaining structural integrity while enabling functional performance in drug delivery applications. The observed peak shifts and new absorptions provide clear evidence of the chemical interactions stabilizing the composite system. Notably, the characteristic BmimBF_4_ peaks (1169, 844, and 753 cm^−1^) remained unchanged, demonstrating the ionic liquid’s stability even in the presence of microbeads. The progressive shifts in the C=O stretching region (from 1712 to 1708 and 1704 cm^−1^) highlight increasing interactions within the hydrogel network, while the persistence of BmimBF_4_ signals underscores its robust integration. These findings demonstrate that the multicomponent hydrogels maintain each constituent’s chemical integrity while forming a cohesive structure through hydrogen bonding, electrostatic interactions, and hydrophobic associations, with the ionic liquid playing a pivotal role in network stabilization. These results are significant for developing functional materials with tailored properties for electrochemical applications.

X-ray diffraction analysis (Figure 2c) revealed that all three nanocomposites exhibited a crystalline structure with a prominent peak at approximately 20°, specifically at 20.48° for PVA-PAA-IL and 20.2° for PVA-PAA-Alg-IL hydrogels. Moreover, the incorporation of microbeads (PVA-PAA-Alg-MBs-IL) did not significantly alter the crystalline structure, maintaining a peak at 20.2°, which suggests minimal disruption to the hydrogel’s intrinsic order. Figure S5b presents the X-ray diffraction pattern of the PVA-PAA-Alg formulation, showing a crystalline structure characterized by a distinct peak at 20.48° (2θ). This diffraction pattern is remarkably similar to that of the PVA-PAA-Alg-IL composite containing ionic liquid. These results demonstrate that the incorporation of ionic liquid into the hydrogel structure does not significantly alter its crystalline properties, maintaining the original structural configuration.

Generally, the results confirm that the microbead–hydrogel composite maintains its structural and electrochemical properties while gaining additional functionality from the incorporated microbeads.

### 2.2. Mechanical Properties of Hydrogels

**Figure 3:**
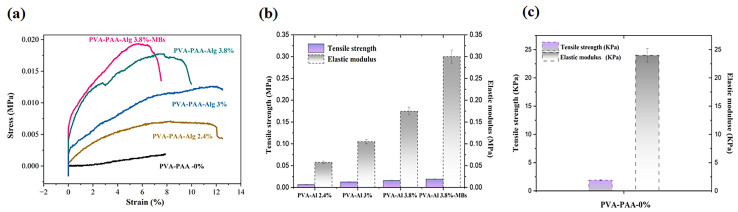
Mechanical characterization of nanocomposite hydrogels: (**a**) Stress–strain curves for PVA-PAA-Alg hydrogels with varying alginate concentrations (0%, 2.4%, 3%, and 3.8%) and PVA-PAA-Alg-MBs hydrogel with 3.8% alginate; (**b**) Comparative tensile strengths and elastic moduli of hydrogels with different alginate concentrations; (**c**) Tensile strengths and elastic moduli of PVA-PAA-0% hydrogel (*n* = 3, mean ± SD).

The evaluation of stretch ability during electrolytic hydrogel preparation revealed the critical influence of alginate concentration on mechanical properties [30]. Systematic analysis of hydrogels with varying alginate contents (2.4%, 3%, and 3.8%) demonstrated a direct concentration–mechanical strength correlation (Figure 3). This study showed that tensile strength increased from 0.0044 ± 0.0003 MPa (SEM = 0.0001) for PVA-PAA-Alg-2.4%, to 0.01 ± 0.005 MPa (SEM = 0.003) for PVA-PAA-Alg-3%, and 0.014 ± 0.001 MPa for PVA-PAA-Alg-3.8% hydrogels. In contrast, the control PVA-PAA-Alg-0% hydrogel showed significantly lower strength (1.9 ± 0.49 kPa, SEM = 0.28) (Figure 3a). This behavior suggests enhanced PVA-PAA chain interactions facilitated by an increasing alginate content.

The optimal 3.8% alginate formulation was selected to prepare hydrogels with mi-crobeads. Comparative analysis showed that the microbead-incorporated hydrogel PVA-PAA-Alg-3.8%–MBs achieved a superior tensile strength of 0.02 ± 0.003 MPa (SEM = 0.002), attributed to the polysaccharide microbeads dual function: maintaining stable em-bedding while forming additional physical and hydrogen bonds with PVA-PAA-alginate chains. Acting as natural crosslinkers, the microbeads reinforce the matrix by restricting chain mobility without compromising elasticity.

**Figure 4:**
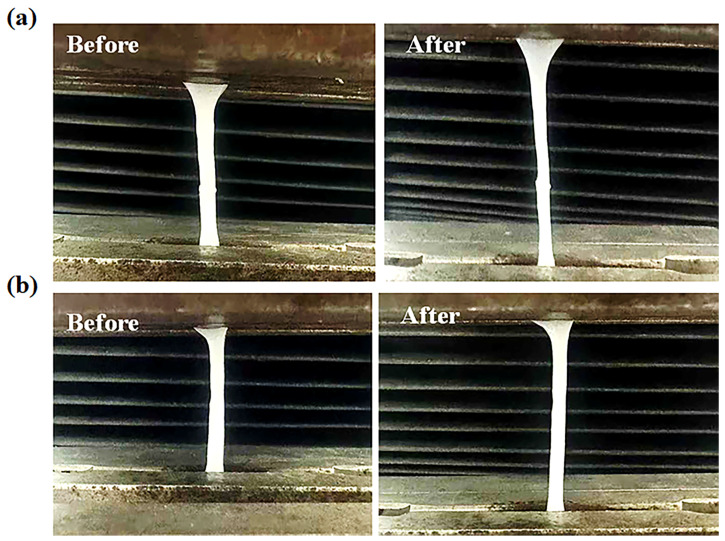
Photographic comparison of PVA-PAA-Alg-3.8% (**a**) and PVA-PAA-Alg-3.8%-MBs (**b**) hydrogels before and after mechanical testing.

Visual analysis (Figure 4) confirmed intact structural homogeneity during defor-mation, with the microbead-enhanced version demonstrating superior mechanical integ-rity under stress. These optimized properties—combining enhanced tensile strength (0.02± 0.003 MPa) with preserved elasticity—establish the composite’s potential for ad-vanced applications. In electrode systems, the material withstands cyclic deformation stresses, while in iontophoresis devices, its stability ensures reliable active substance de-livery under mechanical loads. The microbeads may create supplementary ion transport pathways, potentially enhancing conductivity—a feature requiring further investigation that suggests exciting possibilities for multifunctional optimization. This unique hydrogel with polysaccharide microbeads at a 3.8% alginate (PVA-PAA-Alg-3.8%-MBs) content serves as a versatile platform for electrochemical applications requiring both structural durability and controlled release capabilities.

### 2.3. Adhesive Properties

**Figure 5:**
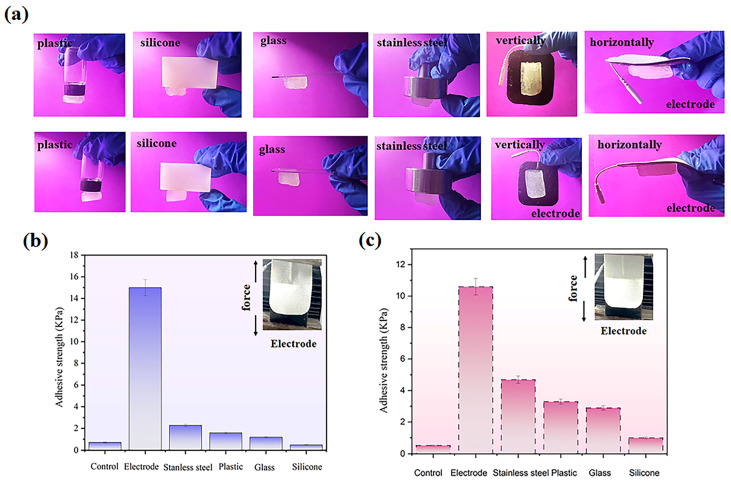
Adhesion properties of electrolyte hydrogels: (**a**) Demonstration of PVA-PAA-Alg-MBs-IL (top) and PVA-PAA-Alg-IL (bottom) hydrogels adhered to various substrates (plastic, silicone, glass, stainless steel, commercial electrodes) in vertical and horizontal orientations. (**b**) Adhesive strength measurements for PVA-PAA-Alg-IL hydrogel. (**c**) Adhesive strength measurements for PVA-PAA-Alg-MBs-IL hydrogel. (*n* = 3, mean ± SD).

The adhesive characteristics of the hydrogel represent a critical parameter governing its interaction with commercial electrodes. To ensure optimal electrical conductivity and functionality throughout the operational period, the hydrogel must maintain robust adhesion to the electrode surface while preserving structural integrity during detachment [31]. Comprehensive adhesion testing (Figure 5a) revealed that both microbead-incorporated and microbead-free hydrogel formulations exhibit excellent interfacial bonding across diverse substrates including plastic, silicone, glass, metal, and commercial electrodes, regardless of orientation.

Quantitative measurements of adhesive strength with electrodes (Figure 5b,c) showed values of 15.0 ± 0.57 kPa (SEM= 0.33) for the microbead-free hydrogel and 10.2 ± 0.62 kPa (SEM = 0.25) for the microbead-loaded variant. These statistically significant interactions with the adhesive layer of commercial electrodes may play an important role in future practical applications. This behavior originates from the synergistic interplay of PVA hydroxyl groups, which mediate reversible substrate bonding, and alginate-PVA chain entanglements that prevent cohesive failure while ensuring residue-free detachment. The microbeads function as neutral structural elements within this network, neither disrupting interfacial interactions nor diminishing the hydrogel’s mechanical resilience.

The developed hydrogel system exhibits balanced adhesion properties within the 10–15 kPa range, making it particularly suitable for electrotherapy applications by delivering reliable bonding while maintaining user comfort during application, as confirmed by test results demonstrating the system’s optimal performance. This adhesion profile ensures secure electrode contact during microcurrent and iontophoresis treatments, guaranteeing stable electrical conductivity. These properties achieve an ideal balance between the need for secure device fixation during procedures and gentle post-treatment removal—a crucial advantage for technologies requiring precise adhesion control.

### 2.4. Conductive and Electrochemical Characterizations

**Figure 6:**
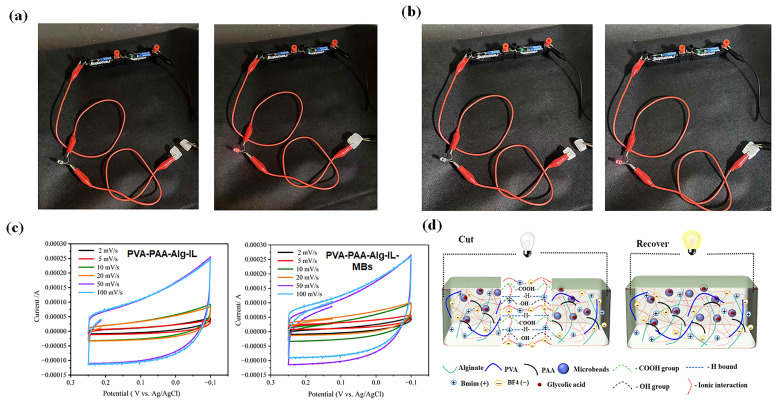
LED integration tests demonstrating conductivity recovery after cutting: (**a**) PVA-PAA-Alg-IL electrolyte hydrogel; (**b**) PVA-PAA-Alg-MBs-IL electrolyte hydrogel with microbeads.; (**c**) Cyclic voltammetry profiles of both hydrogels measured in PBS using a three-electrode system with Ag/AgCl reference; (**d**) Schematic illustration of the cutting-recovery process in hydrogel–LED circuits.

The integration of polymers into electrical systems necessitates a careful evaluation of their electrolytic stability [32]. We systematically assessed this critical parameter through comprehensive conductivity measurements and cyclic voltammetry (CV) analyses of the fabricated electrolytic hydrogels. Using an Ag/AgCl reference electrode system, CV scans conducted across a range of scan rates (2–100 mV/s) demonstrated consistent redox be-havior (Figure 6c), confirming the hydrogels’ structural and electrochemical stability un-der dynamic operating conditions. This characteristic proves particularly vital for appli-cations requiring repeated charge–discharge cycles, such as wearable electronics and transdermal drug delivery systems.

To further verify operational stability, we performed an LED integration test, as schematically shown in Figure 6d, where a red LED maintained continuous illumination even after mechanical recovery (Figure 6a,b), demonstrating preserved ionic conductivity. Complementary recovery experiments (Figure S6) reinforced these findings, showing maintained structural integrity and electrical functionality following deformation. These self-recovery properties align well with established research on resilient conductive hy-drogels [33,34], suggesting strong potential for long-term implementation in flexible bioe-lectronics applications where durable electrolyte performance is essential.

Collectively, these results confirm that the developed hydrogels meet the stringent stability requirements for advanced electrochemical devices while offering the necessary mechanical flexibility for next-generation electrical applications. The unique combination of electrochemical stability, structural resilience, and sustained conductivity under mechanical stress positions these hydrogels as particularly promising candidates for diverse bioelectronic implementations.

Moreover, the Nyquist plots in Figure S7 demonstrate the electrochemical behavior of both hydrogel systems, with distinct high-frequency semicircles characterizing their impedance response. The PVA-PAA-Alginate-IL hydrogel exhibited a substantial ionic conductivity of 8.30 ± 0.48 S/m under standard measurement conditions, as shown in Figure S7a. This favorable conductivity arises from multiple contributing factors including the dissociation of ionic liquid into mobile charge carriers, supplementary charge transport provided by carboxyl groups from the PAA component, and the optimized porous architecture of the alginate matrix which facilitates unimpeded ion movement. Further enhancement was achieved in the PVA-PAA-Alg-MBs-IL formulation incorporating polysaccharide microbeads, which showed a slightly improved conductivity of 9.17 ± 0.55 S/m (Figure S7b). Its conductivity enhancement may stem from the microbeads’ ability to establish interconnected porous networks that promote ion mobility while preserving the material structural integrity, combined with additional proton transfer pathways created by carboxyl functional groups present on the polysaccharide microbeads surfaces.

These results collectively demonstrate effective ionic liquid distribution throughout the polymer matrix and indicate strong potential for these materials in advanced applications requiring both high ionic conductivity and mechanical stability. The preservation of base material properties alongside improved conductivity suggests that these modified hydrogels could be readily implemented in existing applications while providing performance benefits.

### 2.5. Stability of the Hydrogel Under Various Storage Conditions

**Figure 7:**
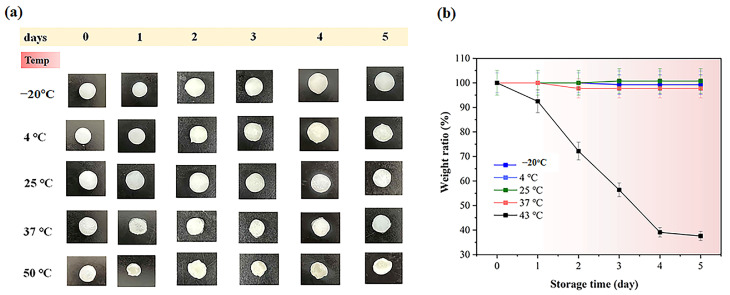
(**a**) Initial photographs of PVA-PAA-Alg-IL hydrogel electrolytes stored for 5 days at different temperatures (−20 °C, 4 °C, 25 °C, 37 °C, and 50 °C). Scale bar: 1 cm; (**b**) weight ratio (%) of the PVA-PAA-Alg-IL hydrogel (*n* = 3, mean ± SD).

**Figure 8:**
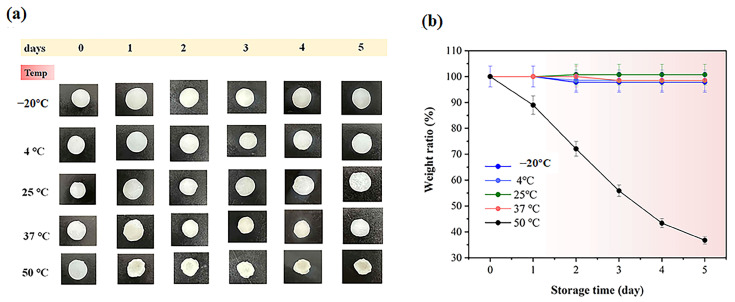
(**a**) Initial photographs of PVA-PAA-Alg-MBs-IL hydrogel electrolytes stored for 5 days at different temperatures (−20 °C, 4 °C, 25 °C, 37 °C, and 50 °C). Scale bar: 1 cm; (**b**) weight ratio (%) of the PVA-PAA-Alg-MBs-IL hydrogel (*n* = 3, mean ± SD.

When studying how different conditions affect cosmetic products, understanding their stability and durability under real-world conditions is crucial [35]. This experiment examined the behavior of hydrogel groups under various temperatures, humidity levels, and changes in conductivity over time. As shown in Figure 7 and Figure 8, which display the temperature-dependent behavior of the hydrogels, the weight ratio (%) values for electrolyte hydrogels on day 5 were 99.0% ± 0.5, 99.5% ± 1.2, 99.7% ± 1.1, 97.5% ± 0.4, and 36.8% ± 0.8 for PVA-PAA-Alg-IL, while for hydrogels with microbeads (PVA-PAA-Alg-MBs), the values were 97.1% ± 0.7, 97.8% ± 0.8, 99.8% ± 1.1, 97.1% ± 1.5, and 36.5% ± 1.8 at temperatures of −20, 4, 25, 37, and 50 °C, respectively. The experiment revealed that at 50 °C, both hydrogels showed significant mass loss due to hydrogel matrix breakdown under high temperatures, while at other temperatures the hydrogel structures remained stable. A similar pattern was observed for non-electrolyte hydrogels (Figures S8 and S9), with particularly stable behavior at 4, 25, and 37 °C, where the hydrogels maintained stability over 5 days. The exception was exposure to 50 °C, where weight ratio (%) values on day 5 were 29.9 ± 0.8% for PVA-PAA-Alg and 38.7% ± 1.8% for PVA-PAA-Alg-MBs. The slight difference in weight ratio % between hydrogels may be due to the protective effect of microbeads in the hydrogel matrix under high temperatures.

**Figure 9:**
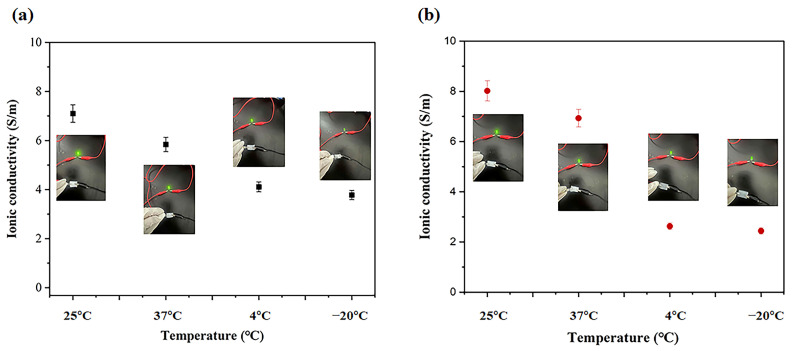
Ionic conductivity of (**a**) PVA-PAA-Alg-IL and (**b**) PVA-PAA-Alg-MBs-IL hydrogel electrolytes measured after 5 days of storage at different temperatures (25 °C, 37° C, 4 °C, and −20 °C) (*n* = 3, mean ± SD).

Here, we also investigated how different conditions affected the conductivity of electrolyte hydrogels after 5 days (Figure 9). Both hydrogels showed similar conductivity trends. At 25 and 37 °C, PVA-PAA-Alg-IL conductivity was 7.1 ± 0.6 S/m and 5.84 ± 0.5 S/m, while PVA-PAA-Alg-MBs-IL showed 8.02 ± 0.72 S/m and 6.93 ± 0.6 S/m—a small decrease from initial values (Figure S7). The slight conductivity reduction at 37 °C (compared to 25 °C) is likely caused by the temporary reorganization of polymer chains at higher temperatures, which slightly slows ion movement. Importantly, this minor decrease does not affect material performance, as values remain well above practical requirements. At lower temperatures (−20 °C and 4 °C), conductivity decreased to 4.11 ± 0.37 S/m and 3.78 ± 0.34 S/m for PVA-PAA-Alg-IL and 2.63 ± 0.24 S/m and 2.44 ± 0.22 S/m for PVA-PAA-Alg-MBs-IL. The particularly low conductivity at −20 °C probably results from partial hydrogel matrix damage under freezing conditions, disrupting ion transport. Despite these changes after 5 days at different temperatures, the maintained conductivity confirms these materials’ suitability for electronic devices and stability under various storage conditions.

**Figure 10:**
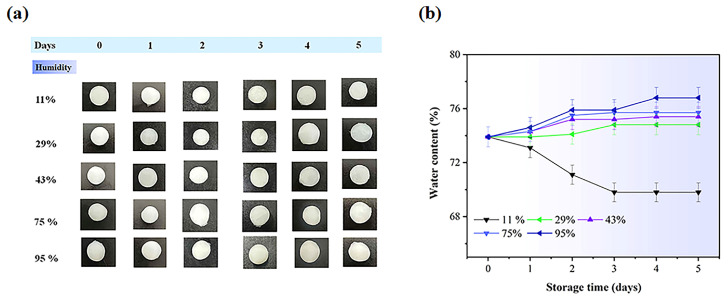
(**a**) Initial photographs of PVA-PAA-Alg-IL hydrogel electrolytes stored for 5 days at different humidity levels (11%, 29%, 43%, 75%, and 95%). Scale bar: 1 cm; (**b**) Water content (%) of PVA-PAA-Alg-IL hydrogels (*n* = 3, mean ± SD).

**Figure 11:**
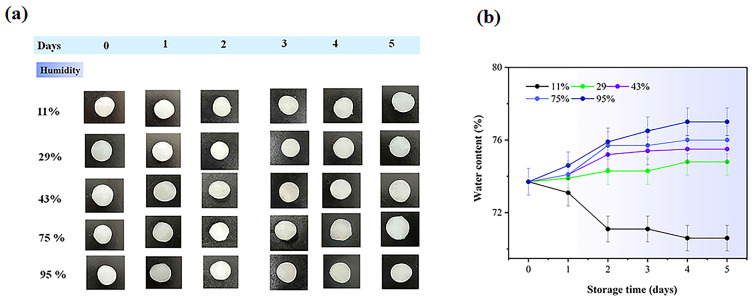
(**a**) Initial photographs of PVA-PAA-Alg-MBs-IL hydrogel electrolytes stored for 5 days at different humidity levels (11%, 29%, 43%, 75%, and 95%). Scale bar: 1 cm; (**b**) Water content (%) of PVA-PAA-Alg-IL hydrogels (*n* = 3, mean ± SD).

Figure 10 and Figure 11 display the water content in electrolyte hydrogels. Both hydrogel types exhibited increased water absorption when humidity rose from 11% to 95%, with particularly notable changes observed on day 5: PVA-PAA-Alg-IL hydrogels increased from 70.91% ± 0.55 to 76.66% ± 0.31%, while PVA-PAA-Alg-MBs-IL showed a similar increase from 69.74% ± 0.30 to 76.87% ± 0.23%. This likely occurs because the ionic liquid creates additional channels between PVA, PAA, and alginate chains, enhancing water absorption at higher humidity. Non-electrolyte hydrogels exhibited similar behavior (Figures S10 and S11), maintaining structural integrity while their water content increased from 11% to 95% under humid storage conditions.

**Figure 12:**
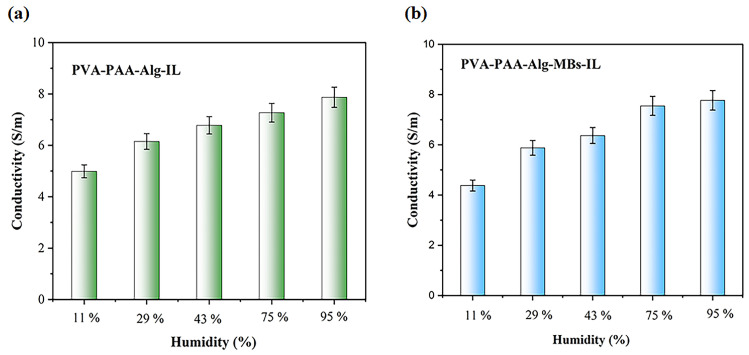
Ionic conductivity of PVA-PAA-Alg-IL (**a**) and PVA-PAA-Alg-MBs-IL (**b**) Hydrogel electrolytes after 5 days of storage under different humidity conditions (*n* = 3, mean ± SD).

Figure 12 shows conductivity changes in electrolyte hydrogels after 5 days at different humidity levels. At 95% humidity, conductivity peaked for PVA-PAA-Alg-IL (7.87 ± 0.83 S/m) and PVA-PAA-Alg-MBs-IL (7.77 ± 0.47 S/m) (Figure 12a,b), as the hydrogel matrix absorbs more water and the ionic liquid facilitates ion transport. This correlation matches the water content results in Figure 10 and Figure 11. At 11% humidity, conductivity decreased to 4.99 ± 0.65 S/m for PVA-PAA-Alg-IL and 4.38 ± 0.5 S/m for PVA-PAA-Alg-MBs-IL. These results demonstrate the excellent stability of electrolyte hydrogels, maintaining conductivity under various storage conditions. The combination of PVA and PAA polymers with ionic liquid (BmimBF_4_) shows synergistic effects, providing stable conductivity that confirms their potential for electronic devices. Similar results have been reported for other PVA- and PAA-based polymer systems with ionic components that maintain conductivity under different temperature and humidity conditions over time [36,37,38,39].

### 2.6. In Vitro Permeability Study

**Figure 13:**
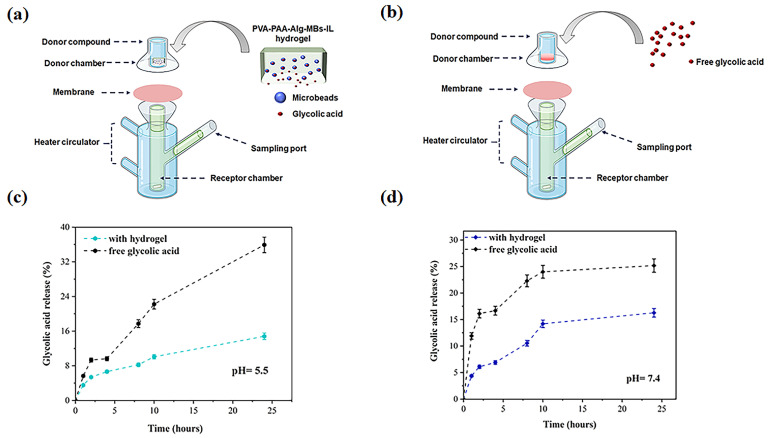
(**a**) Schematic illustration of Franz diffusion cell experimental setup for PVA-PAA-Alg-MBs-IL hydrogel incorporating glycolic acid (GA)-loaded microbeads; (**b**) Free GA solution. Cumulative release profiles of GA: (**c**) Free form and hydrogel matrix at pH 5.5; (**d**) Free form and hydrogel matrix at pH 7.4. (*n* = 3, mean ± SD).

This study results demonstrate significant differences in the release kinetics of glycolic acid (GA) between its free form and the hydrogel-based delivery system (Figure 13). Quantitative analysis revealed substantially higher cumulative release of free GA compared to the hydrogel formulation at both pH 5.5 (35.91 ± 0.10% vs. 14.81 ± 0.07% after 24 h) and pH 7.4 (27.17 ± 0.20% vs. 16.27 ± 0.13%), indicating the pronounced release-retarding effect of the hydrogel matrix. This phenomenon can be attributed to multiple factors including diffusion barriers created by the microbeads, the resistance of the hydrogel network, and the selective permeability of the polyethersulfone membrane. Notably, even free GA showed significant slower release compared to PBS control (Figure S3), demonstrating that the membrane plays a crucial role in modulating release kinetics. The controlled release profile combined with the confirmed absence of cytotoxicity (Figure S12) presents promising opportunities for developing innovative long-acting dermo cosmetic formulations, particularly for implementing gentle peeling protocols with minimal irritation risk. Antimicrobial testing revealed critical material stability properties, with both PVA-PAA-Alg-IL and PVA-PAA-Alg-IL-MBs formulations demonstrating significant bacterial resistance (Figure S13). The hydrogel platform shows the translational potential for daily skincare applications where sustained, low-dose GA delivery is preferred over rapid high-concentration exposure. To advance this delivery system toward clinical application, future studies should evaluate alternative membrane materials with different hydrophilic–lipophilic properties to better match skin permeability. Comprehensive in vivo testing will be necessary to assess bioavailability, skin tolerance, and pharmacokinetics. Additionally, research should explore potential synergistic effects when combining this system with other active ingredients to improve treatment outcomes and broaden its applications.

### 2.7. In Vitro Glycolic Acid Release Under Different Voltaged

**Figure 14:**
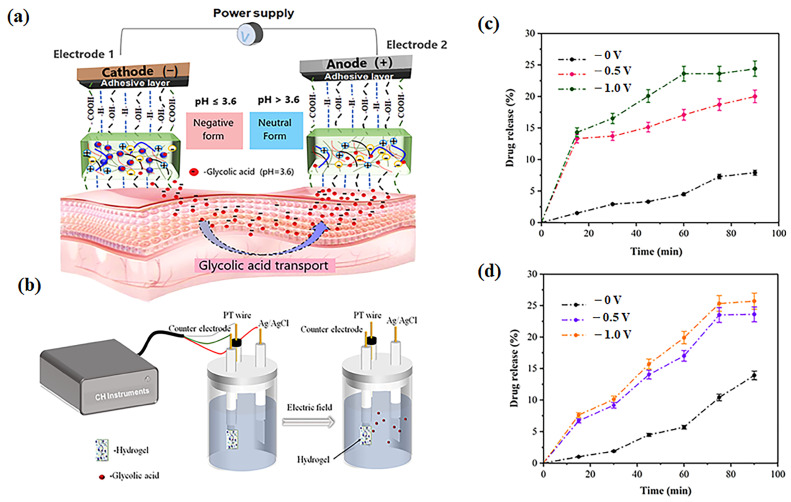
Iontophoretic transdermal delivery mechanism: (**a**) Schematic of iontophoresis device with electrolyte hydrogel patch facilitating glycolic acid (GA) release through cathode-anode electrophoretic migration; (**b**) Integrated ag/AgCl electrode system (CH Instruments) for controlled GA delivery; (**c**) Cumulative GA release profiles from PVA-PAA-Alg-MBs-IL hydrogel in PBS (pH 5.5) under applied voltages (0 V, −0.5 V, −1.0 V); (**d**) Corresponding release profiles in PBS (pH 7.4) under identical electrical stimulation.

The controlled release of glycolic acid from electrolytic hydrogels was investigated using an electrochemical workstation with an Ag/AgCl electrode system under applied voltages of 0 V, −0.5 V, and −1.0 V to simulate iontophoretic conditions (Figure 14). Hydrogels loaded with glycolic acid (pH 3.6) were immersed in PBS solutions at two physiological pH values (5.5 and 7.4) to evaluate pH-dependent release kinetics. Quantitative analysis revealed significantly enhanced release under applied voltages compared to passive diffusion, with cumulative release reaching 20% at −0.5 V and 24.4% at −1.0 V for pH 5.5, while at pH 7.4 the values increased to 23.6% (−0.5 V) and 25.7% (−1.0 V). In contrast, passive release (0 V) yielded only 7.9% at pH 5.5 and 13.9% at pH 7.4, demonstrating clear voltage-dependent transport enhancement. These findings confirm the electrophoretic migration of glycolate anions (COO^−^) toward the anode, consistent with fundamental iontophoresis principles. The sub-quantitative transport efficiency (<100% after 90 min) suggests competing mechanisms including counter-migration of cations toward the cathode creating ionic counterflow, and partial recombination of COO^−^ with protonated carboxyl groups (COOH) near electrode interfaces, observations which align with previous reports of voltage-enhanced drug release from hydrogel matrices [40,41].

This advanced platform combines electrolyte functionality, drug depot capabilities, and uniform current distribution in a single integrated system, representing a significant innovation for pH-sensitive cosmetic formulations requiring controlled release parameters.

## 3. Conclusions

We developed an innovative microbead-based system for optimized glycolic acid (GA) delivery via iontophoresis. The uniformly sized microbeads (1048 ± 140.38 μm) were fabricated from *E. prolifera* polysaccharides and gellan gum for enhanced stability, then incorporated into an iontophoretic electrolyte hydrogel. FT-IR, XRD, and SEM analyses confirmed successful component integration. The hydrogel demonstrated optimal mechanical properties (0.02 ± 0.003 MPa tensile strength) with reliable adhesion and self-healing capabilities, retaining conductivity even post-repair as confirmed by LED testing. Cyclic voltammetry verified maintained conductivity in the microbead-incorporated system. Iontophoretic testing at −1.0 V showed significantly enhanced glycolic acid (GA) permeation compared to passive diffusion. The material exhibited excellent conductivity and stability across varying temperature and humidity conditions, with cytotoxicity assays confirming the hydrogel’s biocompatibility. Franz cell experiments revealed a sustained GA release profile, indicating strong potential for controlled-delivery cosmetic applications. This PVA-PAA-alginate-[Bmim]BF_4_-microbead platform uniquely combines iontophoretic enhancement with prolonged release kinetics, offering an advanced transdermal delivery system for cosmeceutical applications such as skin texture improvement.

## 4. Materials and Methods

### 4.1. Materials and Chemicals

*Enteromorpha prolifera* was collected from the coastal region of Qingdao, China. All chemical reagents were obtained from commercial suppliers: sodium alginate (viscosity: 1000–1200 mPa·s), poly (acrylic acid) (PAA; average Mw ~4,000,000), glycolic acid (98% purity), 1-butyl-3-methylimidazolium tetrafluoroborate ([BMIM][BF_4_]; 97% purity), and biotechnology-grade gellan gum (Mw range: 200–300 kDa) were procured from Shanghai Macklin Biochemical Technology Co., Ltd. (Shanghai, China). Polyvinyl alcohol (PVA) was sourced from Shanghai Aladdin Biochemical Technology Co., Ltd. (Shanghai, China). All aqueous solutions and polymer mixtures were prepared using distilled water to ensure batch-to-batch consistency and minimize potential interference from ionic impurities.

### 4.2. Synthesis of the Electrolyte Hydrogels

A series of polyvinyl alcohol (PVA)–polyacrylic acid (PAA) hydrogels with varying alginate contents were synthesized to optimize the polymer composition. Briefly, 4.5 g of PVA (Mw = 85,000–124,000) was dissolved in 100 mL of distilled water under continuous stirring at 80 °C for 1 h. Separately, 5 g of PAA (Mw = 450,000) was dissolved in 100 mL of distilled water. The PVA and PAA solutions were combined, and sodium alginate was added to achieve final concentrations of 0%, 2.4%, 3%, or 3.8% (*w*/*v*). The mixture was cen-trifuged at 5000 rpm for 5 min to ensure homogeneity, cast into silicone molds, and cross-linked by immersion in 12% (*w*/*v*) CaCl2 for 1 h. All hydrogels were rinsed 3–4 times with distilled water to remove residual salts. This procedure yielded hydrogels with different alginate concentrations, which were coded accordingly: PVA-PAA-Alg-0%, PVA-PAA-Alg-2.4%, PVA-PAA-Alg-3%, and PVA-PAA-Alg-3.8%. The formulation with 3.8% alginate (designated PVA-PAA-Alg-3.8%) was selected for subsequent modifications due to its superior structural integrity. To evaluate the effect of microbeads on the hydrogel matrix, we specifically tested their incorporation into the hydrogel with the highest alginate concentration (3.8%).

For composite hydrogels, 0.01 g of glycolic acid-loaded microbeads (Ps-MBs-Gly) was dispersed in 10 mL of the PVA-PAA-Alg-3.8% precursor solution via mechanical stirring (500 rpm, 10 min). The mixture was crosslinked and washed as described above, yielding the final product labeled PVA-PAA-Alg-3.8%-MBs.

Selected hydrogels (PVA-PAA-Alg-3.8% and PVA-PAA-Alg-3.8%-MBs) were equilibrated in 30% (*w*/*v*) [Bmim][BF_4_] ionic liquid solution for 24 h at 25 °C, followed by rinsing to remove unbound ions, producing PVA-PAA-Alg-IL and PVA-PAA-Alg-MBs-IL, respectively.

The selection of a 3.8% alginate content hydrogel combined with Ps-MBs-Gly microbeads measuring 1078 ± 140.38 μm was driven by their synergistic effects on structural and functional performance. The hydrogel thickness provides sufficient mechanical stability to maintain structural integrity during deformation while accommodating the microbeads without compromising the matrix continuity. This microbead size was selected to balance structural integrity with functional performance, avoiding aggregation (smaller beads) or matrix disruption (larger beads). The combination of hydrogel thickness and microbead size was chosen to maintain mechanical strength while facilitating efficient drug loading and release.

### 4.3. Characterizations of Hydrogels

#### 4.3.1. FT-IR, X-Ray, and SEM Analysis

The chemical functional groups of the hydrogels were characterized using Fourier-transform infrared spectroscopy (FT-IR, Nicolet 5700, Thermo Fisher Scientific, Waltham, MA, USA) with samples prepared as KBr pellets (1:100 sample-to-KBr ratio). Morphological evaluation was performed using scanning electron microscopy (SEM, Quanta 250, FEI, Hillsboro, OR, USA) at an accelerating voltage of 20 kV, with samples sputter-coated with 5 nm gold/palladium to enhance conductivity. Both microbead-incorporated and pure hydrogel formulations were examined to compare their microstructural features. Crystalline properties were analyzed by X-ray diffraction (X’Pert PRO MRD, PANalytical, Almelo, the Netherlands) using monochromatic Cu Kα radiation (λ = 1.5406 Å, 45 kV, 40 mA) with a scanning rate of 2°/min over a 2θ range of 5–80°.

#### 4.3.2. Mechanical Measurements

The mechanical properties of the hydrogels were evaluated through tensile testing using a universal testing machine (WDW-500N, Jinan, Shandong, China). Samples of both hydrogel formulations were subjected to uniaxial tension at a constant crosshead speed of 20 mm/min, with real-time acquisition of stress–strain data. The tests were performed on rectangular specimens with standardized dimensions of 10 mm (length) × 4 mm (width) to ensure consistent results.

#### 4.3.3. Adhesive Measurements

For quantitative assessment, adhesion strength measurements were performed using a universal testing machine (WDW-500N, China) through tensile-adhesion testing. This study employed multiple substrate materials to comprehensively evaluate interfacial bonding properties, including plastic, glass, silicone, stainless steel, and commercial elec-trodes. Standardized hydrogel specimens (10 mm × 10 mm) were compressed onto each substrate for 2 min at ambient temperature (25 °C) to establish adhesive contact prior to testing. Tensile measurements were then conducted at a constant displacement rate of 5 mm/min to determine the adhesive strength.

#### 4.3.4. Storage Stability of Electrolytes Hydrogels Under Temperatures and Hymidity Conditions

To evaluate the storage stability, two groups of hydrogels—non-electrolyte and electrolyte—were prepared in a droplet shape with a diameter of 10 mm and thickness of 5 mm. The hydrogels were subjected to different temperature conditions (−20 °C, 4 °C, 25 °C, 37 °C, and 50 °C) for a period of 5 days to assess their long-term stability. Stability was determined by the daily monitoring of weight retention using the following equation:

Weight ratio = Wt/W_0_ × 100% (1)

where Wt is the weight of the hydrogel after t days of storage and W0 is the initial weight of the hydrogel.

For humidity-controlled experiments, samples were stored for 5 days in desiccators maintained at specific relative humidity (RH) levels using saturated salt solutions: lithium chloride (LiCl) for 11% RH, magnesium chloride (MgCl_2_) for 29% RH, potassium carbonate (K_2_CO_3_) for 43% RH, sodium chloride (NaCl) for 75% RH, and potassium sulfate (K_2_SO_4_) for 95% RH. The humidity conditions were maintained at a constant level throughout the 5-day testing period.

The water content of hydrogels was calculated daily during the 5-day study period using the following equation:

Water content (%) = (Ww − Wd)/Ww × 100%(2)

where Ww denotes the wet weight at each time point and W𝒹 is the dry weight of hydrogel.

#### 4.3.5. The In Vitro Glycolic Acid Release from Electrolyte Hydrogel PVA-PAA-Alg-MBs-IL

The release of glycolic acid (GA) in its free form and from hydrogel formulations was evaluated using modified Franz diffusion cells, following a previously described method with minor modifications [42]. Polyethersulfone synthetic membranes (Pall Corporation Supor^®^, Port Washington, NY, USA, 0.45 μm, 47 mm) were mounted on the cells, with the acceptor compartment filled with phosphate buffer (pH 7.4 or 5.5) under constant stirring at 300 rpm. The system was maintained at 32 °C using a thermostatic bath, with receptor chambers of 6.5 mL volume. For the hydrogel release studies, specially fabricated hydrogel disks (5 mm diameter, 2 mm thickness) containing glycolic acid-loaded microbeads (Ps-MBs-Gly) were applied. In contrast, for the free GA studies, 3 mL of a 3% GA solution was added to the donor chamber. Aliquots (400 µL) were collected from the receptor phase at predetermined intervals (1, 2, 4, 8, 10, and 24 h), with each sample immediately replaced by fresh PBS to maintain sink conditions. The GA concentration in the receptor phase was quantified spectrophotometrically at 220 nm using a pre-established calibration curve. The cumulative percentage of GA release was calculated as follows:

Release percentage = M_t_/M_0_ × 100 (3)

where Mt is the cumulative amount of GA released at time t and M_0_ is the initial GA weight in the microbead–hydrogel composite. This setup enabled a comparative analysis of GA release kinetics from free and hydrogel-incorporated formulations under controlled conditions.

#### 4.3.6. Electrical and Electrochemical Measurements

The electrochemical properties of PVA-PAA-Alg-IL and PVA-PAA-Alg-IL-MBs hydrogels were investigated using cyclic voltammetry (CV) and electrochemical impedance spectroscopy (EIS) measurements performed on a CHI760E electrochemical workstation with a three-electrode system comprising a platinum counter electrode, an Ag/AgCl reference electrode, and the hydrogel as the working electrode, using 0.01 M PBS (pH 7.4) as the electrolyte. For electro-driven drug release studies, a two-electrode system was employed, consisting of a platinum counter electrode and a glycolic acid-loaded PVA-PAA-alginate-MBs -IL hydrogel (pH 3.6) as the working electrode in 10 mL of PBS release medium at pH 7.4 and 5.5. The hydrogel was subjected to sequential electro-stimulation at 0 V, −0.5 V, and −1.0 V for 3 min each, after which 1 mL aliquots were sampled for UV-Vis analysis at 220 nm to determine the glycolic acid concentration before being returned to the release medium. The cumulative release percentage was calculated using equation [43]:

Drug release (%)= (Ct × V)/M0 × 100%(4)

where Cₜ (mg/mL) represents the concentration of glycolic acid released from the hydrogel under electro-stimulation after t minutes, M_0_ (mg) is the initial drug loading content in the hydrogel, and V (mL) is the total volume of the release medium.

Nyquist plots were recorded for PVA-PAA-Alg-IL and PVA-PAA-Alg-MBs-IL hydrogels using a CHI760E workstation (1 Hz-100 kHz) at 25 °C and 50% RH after 10 min hydration to evaluate their electrochemical behavior.

The ionic conductivity of the synthesized hydrogel electrolytes was determined through electrochemical impedance spectroscopy (EIS) measurements. For these tests, hydrogel samples were prepared as square slabs with standardized dimensions of 50 mm × 10 mm × 2 mm (length × width × thickness) to ensure consistent contact area with parallel stainless-steel electrodes. Prior to electrochemical testing, samples underwent a 5-day conditioning period in controlled environmental chambers to assess long-term stability under various conditions. Temperature stability was evaluated at -20 °C, 4 °C, 25 °C, and 37 °C, while humidity stability tests were conducted at relative humidity (RH) levels of 11%, 29%, 43%, 75%, and 95%, maintained using appropriate saturated salt solutions. This comprehensive conditioning protocol allowed for the systematic comparison of environmental effects on electrochemical performance.

The ionic conductivity (σ, S/m) was calculated from impedance measurements using the following fundamental relationship:

(5) $$\sigma \;=\;\frac{t}{Rb\times A}$$

where t is the sample thickness, R_b_ is the bulk resistance obtained from the impedance plot (Ω), and A is the contact area between the sample and electrodes.

### 4.4. Statistical Method

All experimental data are presented as the mean ± standard deviation (SD) from three independent replicates (n = 3). Statistical comparisons were performed using *t*-test in OriginPro 2024 software (OriginLab Corporation, Northampton, MA, USA). Differences were considered statistically significant at *p* < 0.05.

## Data Availability

The data presented in this study are openly available in article.

## Supplementary Materials

The following supporting information can be downloaded at: https://www.mdpi.com/article/10.3390/gels11090682/s1. Scheme S1: (A) Schematic representation of polysaccharide microbead production using a peristaltic pump with different device configurations; (B) SEM micrographs of microbeads fabricated via peristaltic pump under controlled flow rate variations. (C) Uniform microbead distribution in hydrogel with thickness variation; Table S1: pH-dependent swelling behavior of Ps-MBs-Gly microbeads: swelling indices measured at 2, 4, and 10 days; Figure S1: Initial morphology of Ps-MBs-Gly microbeads in pH-varied buffers at (a) 2, (b) 4, and (c) 10 days post-immersion. Figure S2: pH-dependent morphological changes in Ps-MBs-Gly microbeads observed after (a) 2, (b) 4, and (c) 10 days in buffer solutions; Figure S3: Cumulative release of: (a) free glycolic acid and glycolic acid from Ps-MBs-Gly microbeads at pH 5.5; (b) corresponding release at pH 7.4; Figure S4: Glycolic acid release profiles from polysaccharide microbeads. (a) Initial dry-state microbeads of different sizes (scale bar: 2 cm). (b,c) Cumulative glycolic acid release at pH 5.5 and 7.4 over time; Figure S5: (a) FT-IR spectra of non-electrolyte PVA-PAA-Alg nanocomposite hydrogel; (b) XRD patterns of PVA-PAA-Alg nanocomposite; Figure S6: Self-healing properties in the cut and recover stage after 5 min and with a weight test of 77 g after 24 h recover stage for PVA-PAA-Alg-IL and PVA-PAA-Alg-MBs-IL electrolyte hydrogels (a and b); Figure S7: Electrochemical impedance spectroscopy analysis of (a) PVA-PAA-Alg-IL and (b) PVA-PAA-Alg-MBs-IL hydrogel electrolytes measured after 10 min preparation at 25 °C and 50% relative humidity; Figure S8: Initial photographs of PVA-PAA-Alg hydrogel stored for 5 days at different temperatures (−20 °C, 4 °C, 25 °C, 37 °C, and 50 °C). Scale bar: 1 cm; (b) weight ratio (%) of the PVA-PAA-Alg hydrogel (n = 3, mean ± SD); Figure S9: (a) Initial photographs of PVA-PAA-Alg-MBs hydrogel stored for 5 days at different temperatures (−20 °C, 4 °C, 25 °C, 37 °C, and 50 °C). Scale bar: 1 cm; (b) weight ratio (%) of the PVA-PAA-Alg-MBs hydrogel (n = 3, mean ± SD); Figure S10: Initial photographs of PVA-PAA-Alg hydrogel stored for 5 days at different humidity levels (11%, 29%, 43%, 75%, and 95%). Scale bar: 1 cm; (b) water content (%) of PVA-PAA-Alg hydrogels (n = 3, mean ± SD); Figure S11: Initial photographs of PVA-PAA-Alg-MBs hydrogel stored for 5 days at different humidity levels (11%, 29%, 43%, 75%, and 95%). Scale bar: 1 cm; (b) water content (%) of PVA-PAA-Alg-MBs hydrogels (n = 3, mean ± SD); Figure S12: (a) Fluorescence images of PVA-PAA-Alg, PVA-PAA-Alg-MBs, PVA-PAA-Alg-IL, and PVA-PAA-Alg-MBs-IL hydrogels after 1 and 3 days of incubation in the L929 live/dead assay. Scale bar: 250 μm. Relative cell viability of hydrogels after 1 and 3 days of incubation; Figure S13: Antibacterial properties of (1,2) PVA-PAA-Alg and PVA-PAA-Alg-MBs non-electrolyte hydrogels, and PVA-PAA-Alg-IL and PVA-PAA-Alg-IL-MBs electrolyte hydrogels against (a) *E. coli*, (b) *S. aureus*; (c) representative inhibition zones. References [44,45,46] are cited in the Supplementary Materials.
