# Supplementary material for: An Advanced Adhesive Electrolyte Hydrogel Intended for Iontophoresis Enhances the Effective Delivery of Glycolic Acid Via Microbeads
Source: Gels. 2025 Aug 26;11(9):682. doi: 10.3390/gels11090682 (PMC12470250; doi:10.3390/gels11090682)
Supplement: Supplementary file 1 [file gels-11-00682-s001.zip › gels-3754156-supplementary.pdf]

An advanced adhesive electrolyte hydrogel intended for iontophoresis enhances the effective delivery of glycolic acid via microbeads

Mariia Kazharskaia 1,2, †, Yu Yu 1,2, † and Liu Chenguang 1, *

1 College of Marine Life Sciences, Ocean University of China, No.5 Yushan Road, Qingdao 266003, China; marikazharskay@mail.ru

2 Qingdao Youdo Bioengineering Co., Ltd., No.175 Zhuzou Road, Qingdao 266101, China; yuyu@youdo-bio.com

* Correspondence: liucg@ouc.edu.cn

† These authors contributed equally to this work.

Supporting Information

1. Experimental Procedures

1.1 Preparation of polysaccharides microbeads through peristaltic pump

Preparation of polysaccharide microbeads with different sizes using a ZP300 peristaltic pump (LEIRONG) with a flow rate range of 90-320 mL/min equipped with a single nozzle configuration with 3.6 mm inlet and 2.5 mm outlet diameters (Scheme S1A). This experiment demonstrated that by adjusting the flow rates to 90, 150, 270, and 320 mL/min - particularly by increasing them - microbeads were obtained with controlled sizes of 1411 ± 51 μm, 945 ± 45 μm, 750 ± 45 μm, and 652 ± 74 μm respectively, as shown in Scheme S1B. The results clearly indicate that both flow rate adjustment and nozzle selection play crucial roles in determining the final size distribution of the polysaccharide microbeads, allowing for precise control over their dimensions. This method provides a reliable and tunable approach for producing

microbeads with specific size characteristics required for different experiments.

Initially, water-soluble polysaccharides were extracted from Enteromorpha prolifera using the hot water extraction method [44]. Subsequently, 8 grams of the extracted polysaccharides were dissolved in 100 ml of deionized water and heated at 60°C for 1 hour with constant stirring. In parallel, 2.5 grams of gellan gum were dissolved in 100 ml of deionized water at 90°C for 1 hour. The two solutions were then combined and mixed thoroughly for 10 minutes to achieve a homogeneous mixture. For the continuous phase, a 10% (w/v) $CaCl_2$ solution was prepared. Then under pump polysaccharides solution were dropped to 10% $CaCl_2$ solution through nozzle and keep for 1 h for crosslinking process.

Similarly, microbeads containing glycolic acid (Gly) were prepared. For this purpose, 1 gram of glycolic acid was dissolved in 100 mL of water at pH 3.6. Upon complete dissolution, 2.5 grams of gellan gum were added to 50 mL of the solution, followed by the addition of 50 mL of the prepared glycolic acid solution (pH 3.6). The microbeads were then formed in a continuous $CaCl_2$ (10%) phase, accordingly using pump. The resulting polysaccharide microbeads were washed 3-4 times with distilled water to remove residual reagents, then dried at 40°C for 24 hours. These microbeads were labeled as Ps-MBs (without glycolic acid) and Ps-MBs-Gly (with glycolic acid), respectively.

2. Characterization of prepared microbeads

2.1 FT-IR, X-ray and SEM analysis

For SEM analysis, polysaccharide microbeads (Ps-MBs) and containing glycolic acid (pH=3.6, Ps-MBs-Gly) were fixed in a sample holder and coated with a gold layer for 5 minutes using an S150 sputter coater. The samples were then transferred to the autosampler of a scanning electron microscope (SEM, Quanta 250, FEI, USA). For FTIR analysis (Nicolet 5700, Thermo Fisher Scientific, USA), samples were scanned in the range of 4000–500 cm^{-1}. X-ray diffractograms were acquired using a Siemens D5000 diffractometer with Cu–Kα radiation (40 kV, 30 mA).

2.2 Swelling activity

For the swelling test, 0.06 g of two kind of polysaccharide microbeads were immersed in PBS solutions at different pH values (1.5, 5.5, and 10.5). The swollen microbeads were carefully removed at predetermined time intervals (2, 4, and 10 days) and weighed immediately after blotting excess buffer solution. The swelling degree (S) was calculated using the equation (S1):

$$\text{Swelling degree g/g} = \frac{\text{mt}-\text{mg}}{\text{mg}} \quad \text{(S1)}$$

where mt(g) represents the mass of swollen microbeads at time *t* (days) and *mg* (g) is the mass of dried microbeads. After measurements, the samples were collected, rinsed with water, and photographed (Figure S1)and collected in Table S1, followed by SEM analysis to examine morphological changes in the microbeads after exposure to different pH buffer solutions over varying time periods (Figure S2).The SEM observations provided detailed insights into the structural modifications of the microbeads under different pH conditions and swelling durations. This comprehensive characterization approach allowed for systematic evaluation of the microbeads' stability and swelling behavior in physiologically relevant pH environments, demonstrating their potential for controlled release applications. The combination of gravimetric analysis, visual documentation, and electron microscopy provided a robust assessment of the microbeads performance across different experimental conditions.

2.3 Cumulative release

The drug release assay for glycolic acid-loaded microbeads and pure glycolic acid was performed separately at pH 5.5 and pH 7.4 according to previous work with some modifications [45]. Phosphate buffer solution (PBS), which mimics skin pH conditions, was used with the dialysis bag technique (Himedia, Dialysis Membrane-110 with a molecular weight cutoff of 12,000-14,000 kDa). Dialysis bags containing 200 mg of microbeads in 10 mL of PBS (pH 5.5 or 7.4) were placed in 500 mL of PBS (pH 5.5 or 7.4) maintained at 37±1°C. Samples (2 mL) were withdrawn at predetermined time intervals (30, 60, 90, 120, 150, 180, and 210 minutes) and replaced with an equal

volume of fresh PBS buffer. The concentrations of both pure glycolic acid and glycolic acid released from the microbeads were analyzed using a UV-Visible spectrophotometer at 220 nm, with the percentage cumulative release calculated using a standard calibration curve method. The methodology was equally applicable to microbeads of varying diameters (1411±51 - 652±74 μm) fabricated using a peristaltic pump system (Scheme S1).

Characterization of hydrogels

3.1 Self-healing properties

To examine their self-healing behavior, the hydrogels were cut into two parts, and the sections were immediately brought into contact. The healed hydrogels were then stretched under a 77-gram metallic weight. Optical images of the process were captured using a Canon EOS 80D digital camera.

3.2 Antibacterial activity

The antibacterial properties of the hydrogels were evaluated using the agar disc-diffusion method against Gram-positive Staphylococcus aureus and Gram-negative Escherichia coli. The study was conducted according to established protocols with modifications [46]. All prepared hydrogels were cut into cubic specimens (5 × 5 mm) and placed onto Mueller-Hinton agar plates inoculated with 0.1 mL of bacterial suspension (24-hour cultures of S. aureus or E. coli). The hydrogel samples were positioned on the inoculated agar surface within 5-10 minutes after bacterial seeding. The plates were then incubated at 37°C for 24 hours. Following incubation, the inhibition zones surrounding each hydrogel disk were measured in triplicate using calipers, with results expressed as mean ± standard deviation.

3.3. Cytotoxicity study. Live/dead cell staining

The cytocompatibility of non-electrolyte hydrogels (PVA-PAA-Alg, PVA-PAA-Alg-MBs) and electrolyte hydrogels (PVA-PAA-Alg-IL, PVA-PAA-Alg-MBs-IL) was evaluated using L929 fibroblasts over 1 and 3 days.

For cytotoxicity assessment, accurately weighed hydrogel specimens were sterilized via bilateral UV exposure (40 min per side). The sterilized samples were then fully

immersed in complete growth medium (DMEM supplemented with 10% fetal bovine serum) at a standardized ratio of 100 mg material per mL medium. The mixture was incubated under controlled conditions (37°C, 5% CO_2) for 72 hours to produce conditioned extracts. Following incubation, all extracts were filtered through 0.22 μm membranes to ensure sterility.

In parallel, L929 fibroblasts in logarithmic growth phase were harvested by trypsinization, counted, and plated in 96-well culture plates at a density of 6×10^3 cells per well. After allowing 24 hours for cell attachment under standard culture conditions (37°C, 5% CO_2), the medium was replaced with the prepared hydrogel extracts for subsequent viability assessment.

After 24 hours of extract treatment, cell viability was assessed. Live and dead cells were stained with Calcein-AM (green) and propidium iodide (red) for 30 minutes at 37°C, then imaged using a confocal microscope (Nikon).

Cell viability was quantitatively assessed using the CCK-8 assay (Dojindo, Japan). Following incubation with hydrogel extracts for 1 and 3 days (37°C, 5% CO_2). The optical density (OD) of each well was measured at 450 nm. Cell viability was calculated as follows:

$$\text{Viability} = \frac{\text{OD sample} - \text{ODblank}}{\text{OD control} - \text{OD blank}} \times 100\% \tag{S2}$$

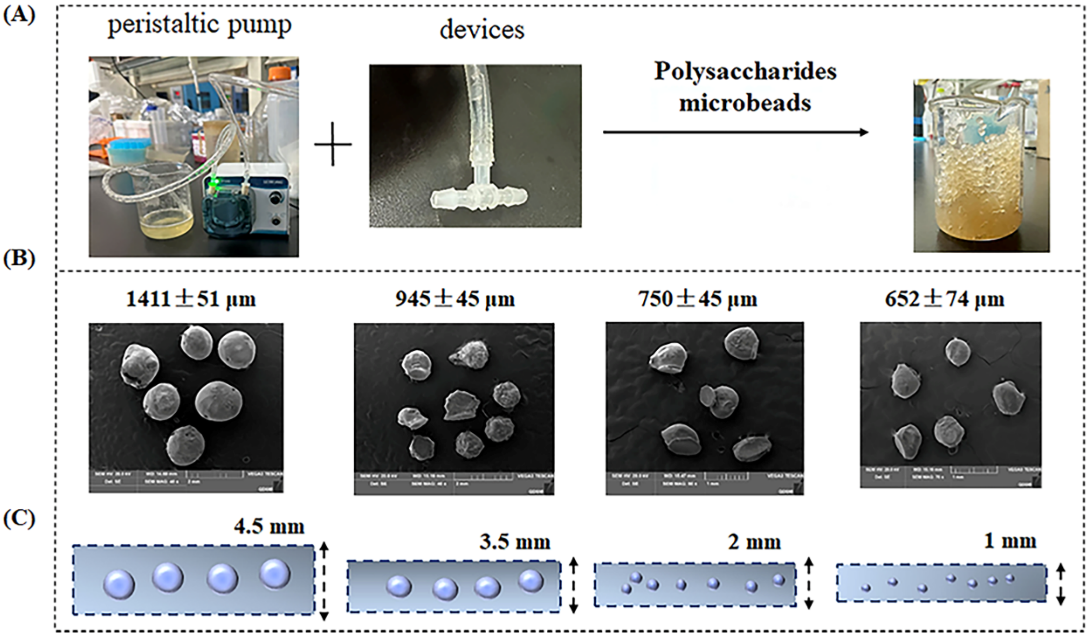

Scheme S1. (A) Schematic representation of polysaccharide microbead production using a peristaltic pump with different device configurations; (B) SEM micrographs of microbeads fabricated via peristaltic pump under controlled flow rate variations. (C) Uniform microbead distribution in hydrogel with thickness variations.

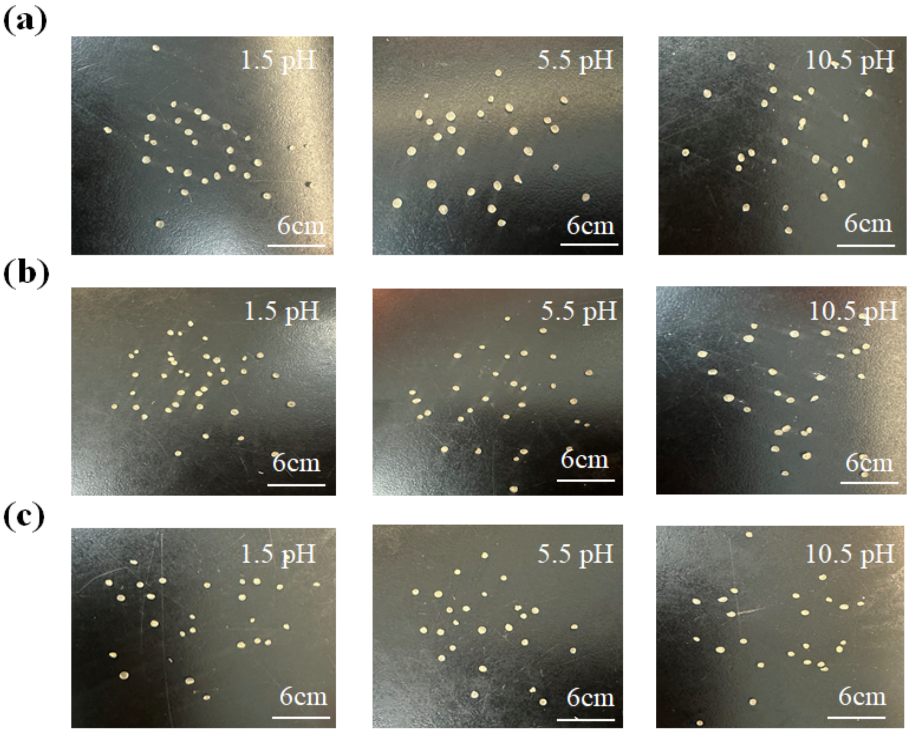

Figure S1. Initial morphology of Ps-MBs-Gly microbeads in pH-varied buffers at (a) 2, (b) 4, and (c) 10 days post-immersion.

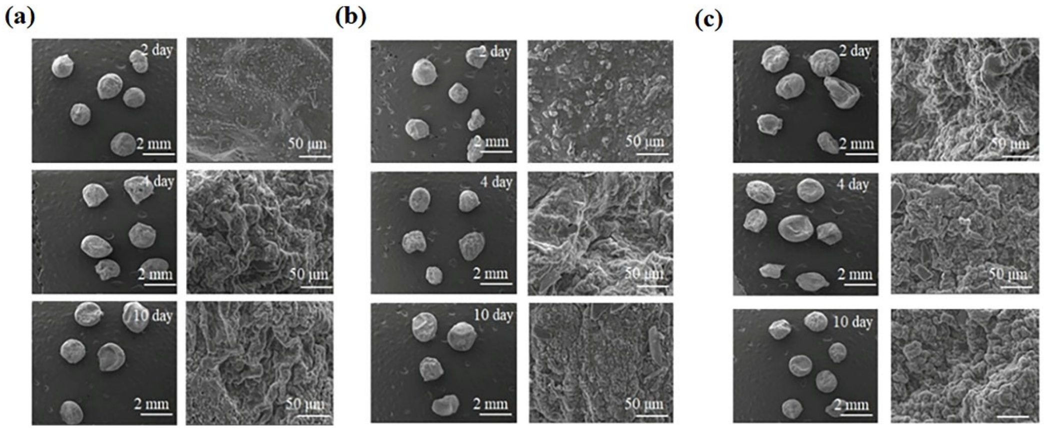

Figure S2. pH-dependent morphological changes in Ps-MBs-Gly microbeads observed after (a) 2, (b) 4, and (c) 10 days in buffer solutions.

Table S1. pH-dependent swelling behavior of Ps-MBs-Gly microbeads: swelling indices measured at 2, 4, and 10 days. (n=3, mean ± SD)

pH/Day	2	4	10
**1.5**	0.13 ± 0.01	0.14 ± 0.005	0.26 ± 0.005
**2.5**	0.12 ± 0.005	0.14 ± 0.00	0.28 ± 0.005
**3.5**	0.12 ± 0.005	0.13 ± 0.005	0.29 ± 0.005
**4.5**	0.14 ± 0.005	0.14 ± 0.01	0.30 ± 0.005
**5.5**	0.15 ± 0.005	0.15 ± 0.01	0.31 ± 0.01
**6.5**	0.16 ± 0.01	0.17 ± 0.05	0.33 ± 0.01
**7.5**	0.16 ± 0.01	0.17 ± 0.005	0.32 ± 0.01
**8.5**	0.1 6 ± 0.005	0.19 ± 0.00	0.34 ± 0.005
**9.5**	0.17 ± 0.01	0.19 ± 0.00	0.33 ± 0.005
**10.5**	0.17 ± 0.005	0.19 ± 0.01	0.33 ± 0.02

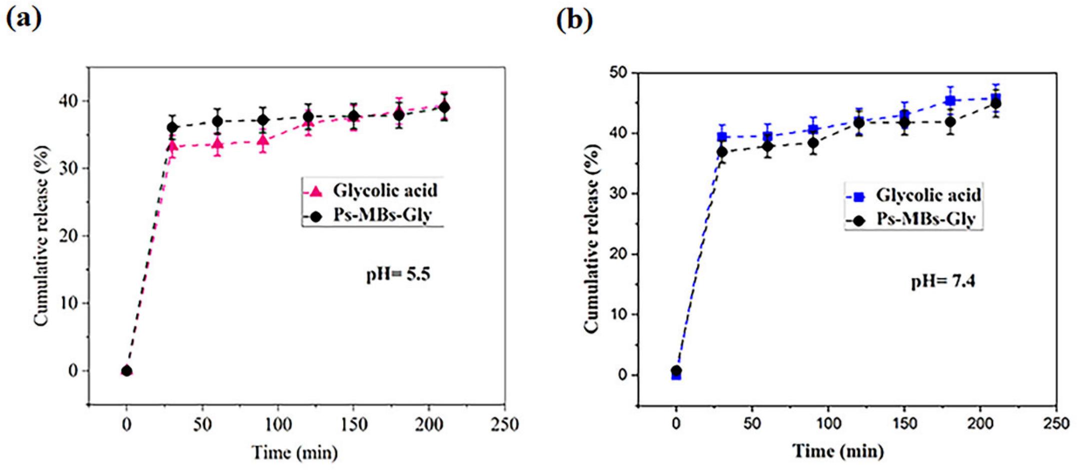

Figure S3. Cumulative release of: (a) free glycolic acid and glycolic acid from Ps-MBs-Gly microbeads at pH 5.5; (b) corresponding release at pH 7.4.

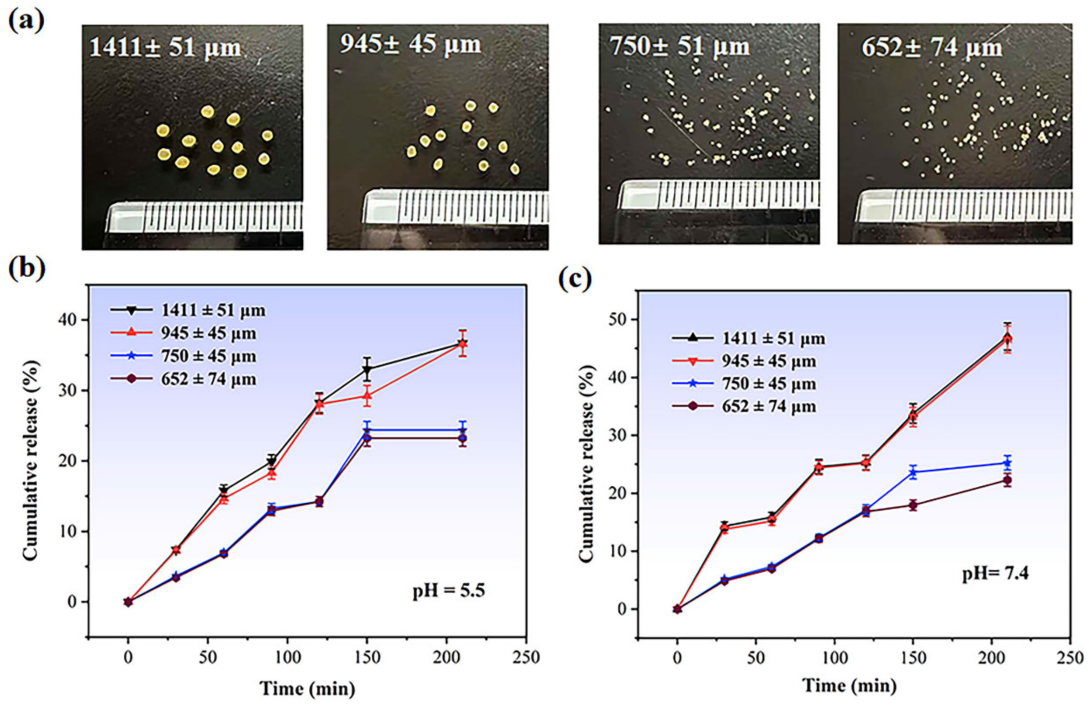

Figure S4. Glycolic acid release profiles from polysaccharide microbeads. (a) Initial dry-state microbeads of different sizes (scale bar: 2 cm). (b, c) Cumulative glycolic acid release at pH 5.5 and 7.4 over time (n=3, mean ±SD).

3. Results and discussion

3.1 Characterizations of PVA-PAA-Alg hydrogel formulation

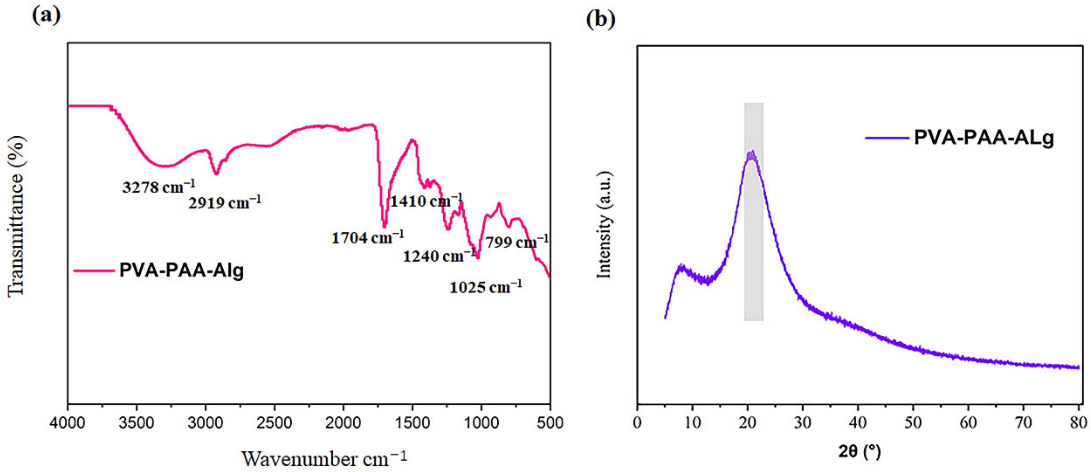

Figure S5. (a) FT-IR spectra of non- electrolyte PVA-PAA-Alg nanocomposite hydrogel; (b) XRD patterns of PVA-PAA-Alg nanocomposite

4.2 Self-healing properties of electrolyte hydrogels

Electrolyte hydrogels exhibited notable self-healing properties, as illustrated in Figure S5. After 24 hours, the hydrogels demonstrated strong cohesive strength, supporting a weight of 77 grams (Figure S5b), even in the presence of embedded polysaccharide microbeads.

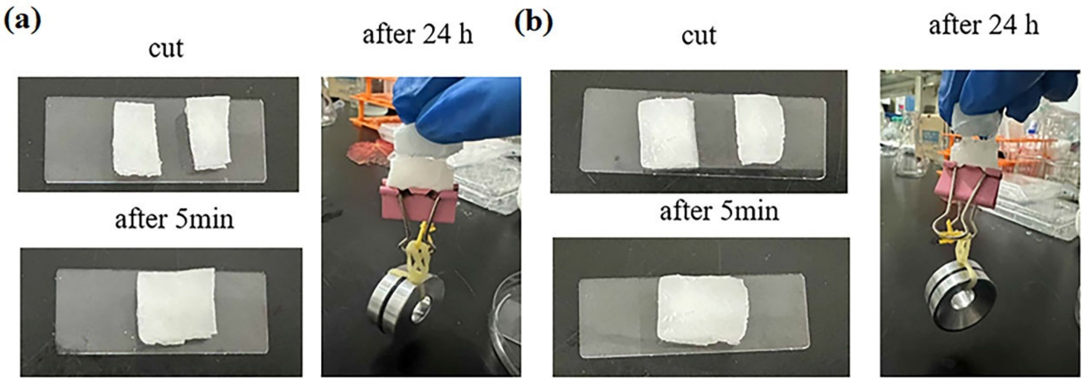

Figure S6. Self –healing properties in cut and recover stage after 5 min and with a weight test of 77 grams after 24 h recover stage for PVA-PAA-Alg-IL and PVA-PAA-Alg-MBs-IL electrolyte hydrogels (a and b)

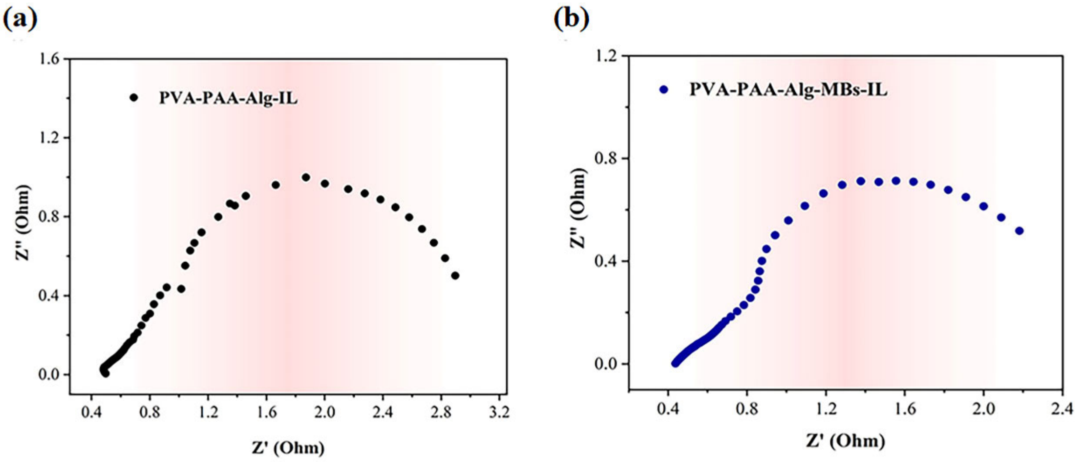

Figure S7 Electrochemical impedance spectroscopy analysis of (a) PVA-PAA-Alg-IL and (b) PVA-PAA-Alg-MBs-IL hydrogel electrolytes measured after 10 minutes preparation at 25°C and 50% relative humidity

4.3. Storage stability of non-electrolyte hydrogels under different temperature conditions

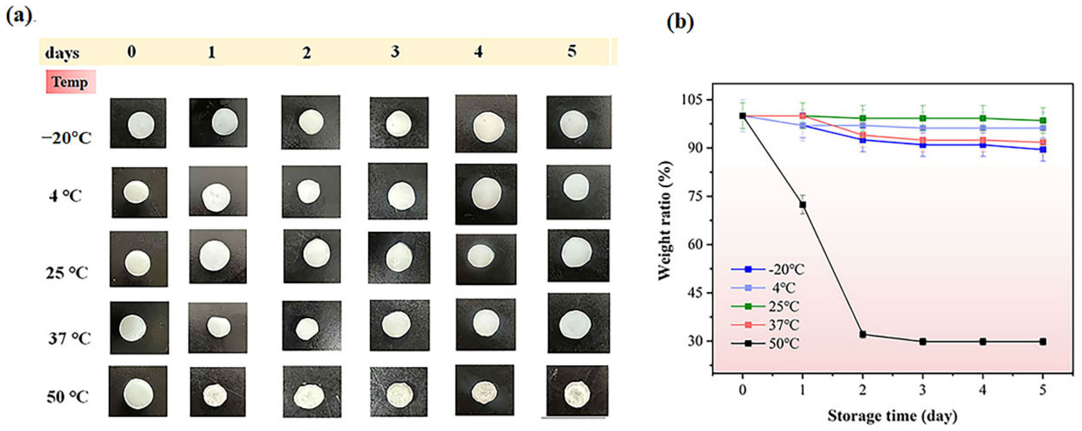

Figure S8 (a)Initial photographs of PVA-PAA-Alg hydrogel stored for 5 days at different temperatures (−20°C, 4°C, 25°C, 37°C, and 50°C). Scale bare:1 cm; (b) Weight ratio (%) of the PVA-PAA-Alg hydrogel (n = 3, mean ± SD)

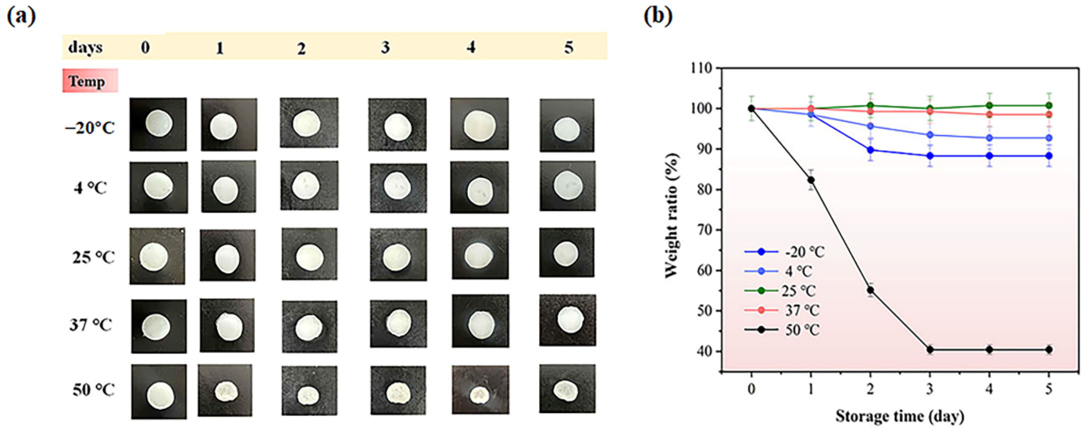

Figure S9 (a) Initial photographs of PVA-PAA-Alg-MBs hydrogel stored for 5 days at different temperatures (−20°C, 4°C, 25°C, 37°C, and 50°C). Scale bare:1 cm; (b) Weight ratio (%) of the PVA-PAA-Alg-MBs hydrogel (n = 3, mean ± SD)

4.4 Storage stability of non-electrolyte hydrogels under varied humidity conditions

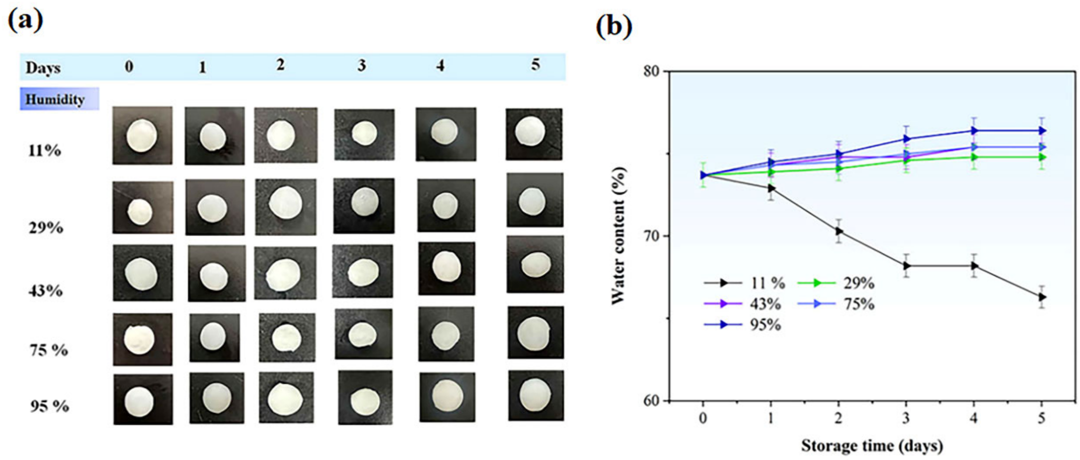

Figure S10. (a)Initial photographs of PVA-PAA-Alg hydrogel stored for 5 days at different humidity levels (11%, 29%, 43%, 75%, and 95%). Scale bare:1 cm; (b) Water content (%) of PVA-PAA-Alg hydrogels (n = 3, mean ± SD)

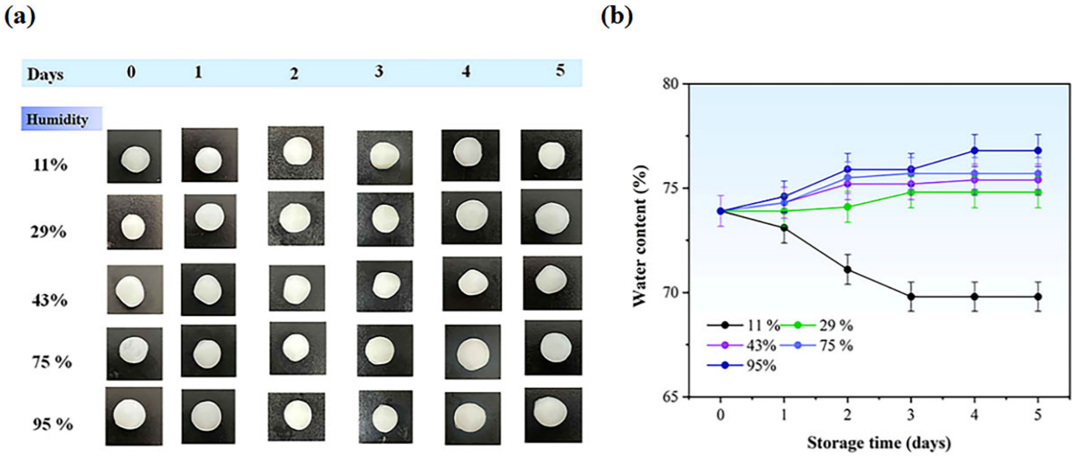

Figure S11. (a)Initial photographs of PVA-PAA-Alg-MBs hydrogel stored for 5 days at different humidity levels (11%, 29%, 43%, 75%, and 95%). Scale bare:1 cm; (b) Water content (%) of PVA-PAA-Alg-MBs hydrogels (n = 3, mean ± SD)

4.5 Cytotoxicity evaluation of synthesized hydrogels

Confocal microscopy (Figure S12a) confirmed excellent cell viability, with L929 fibroblasts maintaining normal morphology and density after 1- and 3-day hydrogel exposure. CCK-8 assays (Figure S12b) quantitatively supported these findings, showing >80% viability for all hydrogel formulations. Together, these results demonstrate the materials' non-toxic nature and biocompatibility, meeting essential

requirements for cosmetic applications involving prolonged skin contact. Combined with their controlled-release properties, these hydrogels show strong potential for therapeutic cosmeceutical products requiring sustained ingredient delivery.

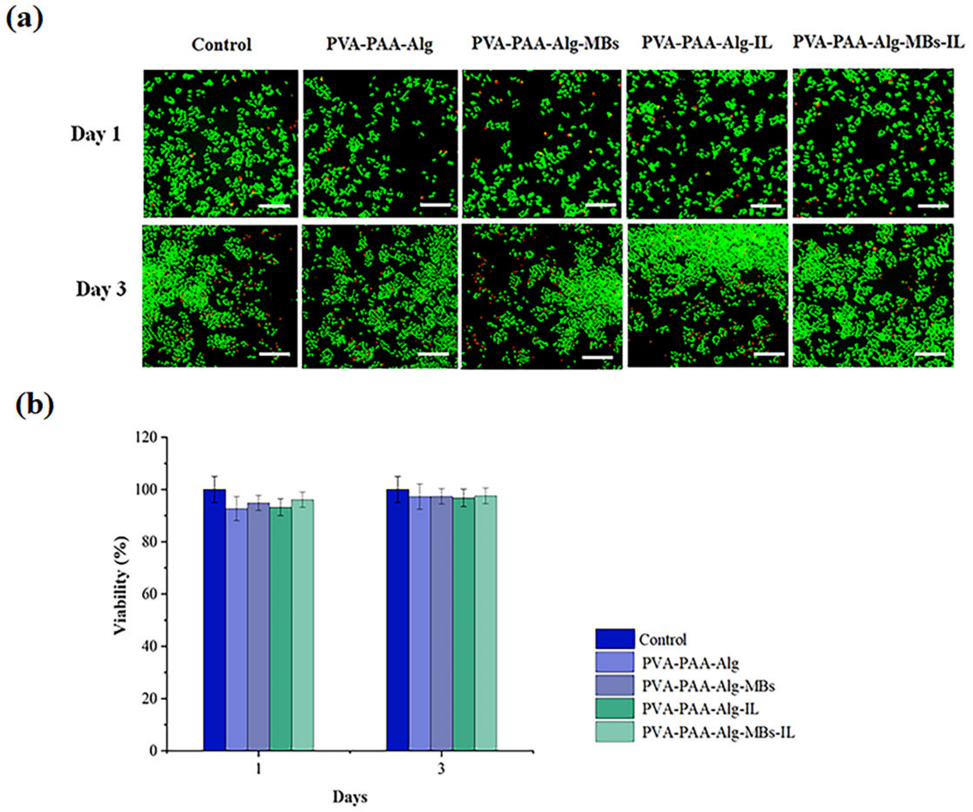

Figure S 12 (a) Fluorescence images of PVA-PAA-Alg, PVA-PAA-Alg-MBs, PVA-PAA-Alg-IL, and PVA-PAA-Alg-MBs-IL hydrogels after 1 and 3 days of incubation in the L929 live/dead assay. Scale bar: 250 μm. (b) Relative cell viability of hydrogels after 1 and 3 days of incubation.

4.6 Antibacterial activity of prepared hydrogel

Antimicrobial testing revealed critical material stability properties, with both PVA-PAA-Alg-IL and PVA-PAA-Alg-IL-MBs formulations demonstrating significant bacterial resistance (Figure S13). Against E. coli, the basic hydrogel formulation exhibited an inhibition zone of 8.6 ± 2.08 mm, while the microbead-containing variant showed enhanced activity (12 ± 3 mm). For S. aureus, comparable inhibition was observed with 8.6 ± 2.08 mm for the basic hydrogel and 7.6 ± 1.2 mm for the microbead-incorporated version, confirming broad-spectrum antimicrobial properties essential for cosmetic product preservation. These antibacterial characteristics,

combined with the controlled release performance and electrical conductivity, position these hydrogels as promising candidates for development into sterile, active-ingredient delivery systems for dermatological applications requiring both microbial protection and enhanced transdermal transport.

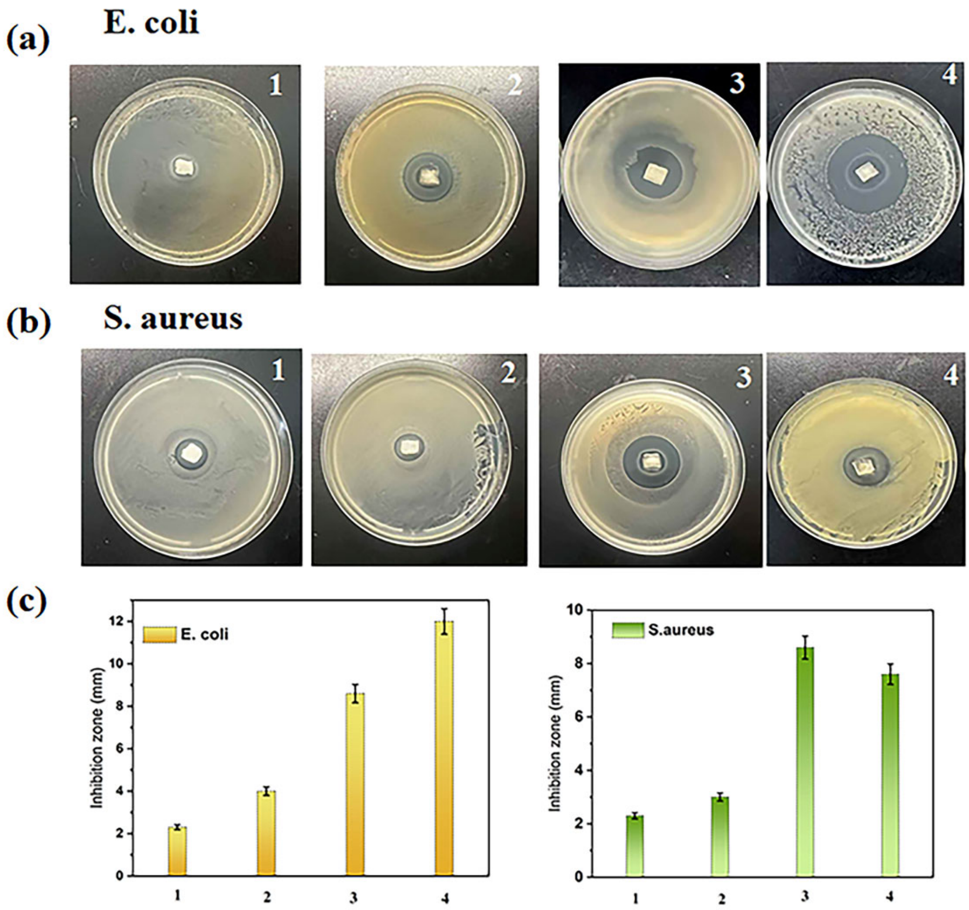

Figure S13. Antibacterial properties of (1,2) PVA-PAA-Alg and PVA-PAA-Alg-MBs non-electrolyte hydrogels, and PVA-PAA-Alg-IL and PVA-PAA-Alg-IL-MBs electrolyte hydrogels against: (a) E. coli, (b) S. aureus; (c) representative inhibition zones.
